# BlinkFusion: modular platform quantifying labeling efficiency and photophysics in regular and super-resolution fluorescence microscopy

**DOI:** 10.3389/fbinf.2026.1817759

**Published:** 2026-05-28

**Authors:** Alejandro Salgado, Nada Naguib, Ulrich B. Wiesner, Alba Ávila

**Affiliations:** 1 Department of Electrical and Electronics Engineering, Universidad de Los Andes, Bogotá, Colombia; 2 Meinig School of Biomedical Engineering, Cornell University, Ithaca, NY, United States; 3 Department of Materials Science and Engineering, Cornell University, Ithaca, NY, United States; 4 Department of Design Tech, Cornell University, Ithaca, NY, United States

**Keywords:** blinking statistics, degree of labeling, fluorescence microscopy, single molecule localization microscopy, stochastic optical resolution microscopy

## Abstract

Quantitative analysis in fluorescence microscopy presents challenges: most open-source Single Molecule Localization Microscopy toolkits emphasize visualization over downstream metrics, and practitioners must iteratively juggle sample preparation variables (e.g., labeling density) with acquisition parameters (e.g., photoswitching conditions) once imaging is underway, obscuring cause effect and slowing optimization. We address this gap with BlinkFusion, a modular, open-source Python platform that unifies filament labeling efficiency and STORM photophysics in a single, reproducible workflow. The system ingests image stacks, extracts metadata, and runs two complementary pipelines: (i) a confocal/filament branch that applies ridge guided ROI selection and Stretching Open Active Contours (SOACs) to quantify degree of labeling (DOL) and morphometrics, providing pre-STORM feedback on staining quality; and (ii) a STORM branch that merges localizations into molecules and computes duty cycle, survival fraction, photon yields, and switching cycles within a quasi-equilibrium window for fair cross dataset comparison. An interactive dashboard enables side by side dataset review, rapid parameter sweeps, and immediate reprocessing. On nanobody labeled tubulin, the filament pipeline automatically captures expected trends in continuity, contrast, and intensity across preparation and illumination settings; on Cy5 benchmarks, the STORM pipeline reproduces literature photophysics within 20% under matched conditions, while reducing peak CPU/heap demand and manual effort. A streamlined DOL workflow cuts processing time versus prior manual practice. BlinkFusion therefore links structural and photophysical readouts to deliver immediate, quantitative feedback and a practical path towards real time experimental optimization.

## 1 Introduction

Fluorescence microscopy has transformed life and materials sciences by enabling analysis of structures far beyond the resolution limits of conventional light microscopy. This technique excels through its exceptional molecular specificity and superior signal to noise ratio, achievements made possible by significant advances in both detector technology and fluorescent labeling chemistry (Toseland, 2013; Suzuki et al., 2007). Standard fluorescence microscopy systems incorporate excitation and emission filters, dichroic mirrors, and highly sensitive detection systems, including Electron Multiplying Charge-Coupled Device (EMCCD) cameras, to enable accurate fluorescence detection (Combs, 2010). This approach has become essential for investigating cellular behavior and biochemical phenomena in both living systems and engineered materials (Renz, 2013; Bradbury and Evennett, 1996; Hickey et al., 2021; Tosi et al., 2022).

A major breakthrough in this field is Single Molecule Localization Microscopy (SMLM), which overcomes the diffraction limit of conventional fluorescence microscopy by exploiting the photophysical properties of individual fluorophores Lelek et al. (2021). While conventional optical imaging is typically limited to resolutions of approximately 200–300 nm, SMLM achieves spatial resolutions below 10 nm under optimal conditions (Valli et al., 2021; Khater et al., 2020). A prominent technique within SMLM, Stochastic Optical Reconstruction Microscopy (STORM), enables high resolution imaging and provides insight into cellular nanostructure (Xu et al., 2017; Rust et al., 2006; Schermelleh et al., 2010). STORM reconstructs images by precisely localizing fluorophores that are stochastically switched on and off using photochemical switching (Small and Stahlheber, 2014). This approach yields resolution improvements of over an order of magnitude compared to diffraction limited methods in fluorescence microscopy. However, the buffers and imaging conditions required for STORM introduce limitations, including photobleaching, cytotoxic effects associated with thiol based, non-physiological buffers, which can compromise sample viability and limit long term live cell imaging (Wäldchen et al., 2015; Wang et al., 2025).

Most general purpose SMLM analysis tools enable accurate fluorophore localization and high quality image reconstruction but remain limited in their ability to provide comprehensive quantitative insight, typically reporting only basic blinking statistics and lacking functionality for systematic dataset comparison or robust characterization of switching behavior (Dankovich and Rizzoli, 2021). A comparison of widely used open-source SMLM software is presented in Table 1, Xu et al. (2017). Platforms such as ThunderSTORM, Picasso, and SMAP offer powerful localization and reconstruction capabilities, but their focus remains on visualization and detection accuracy rather than downstream quantitative analysis. Recent approaches incorporating automation and machine learning, such as AutoDS and Quality Control Maps (QCM), primarily reduce manual parameter tuning during localization rather than enabling comprehensive, user friendly quantitative workflows (Saguy et al., 2025; Mailfert et al., 2025).

**TABLE 1:** Open-source software for SMLM analysis.

Software designation	Functions	Year	References
ThunderSTORM	Pre, Pro, QA	2014	Ovesný et al. (2014)
Picasso	Pre, Pro, QA	2017	Schnitzbauer et al. (2017)
rapidSTORM	Pro, QA	2012	Wolter et al. (2012)
SMAP	Pre, Pro, QA	2020	Ries, (2020)
AutoDS	Pre, Pro	2025	Saguy et al. (2025)
QCM	Pre, Pro, QA	2025	Mailfert et al. (2025)
LAMA	Pro, QA	2016	Malkusch and Heilemann, (2016)
GraspJ	Pro, QA	2012	Brede and Lakadamyali, (2012)

High quality open-source software for STORM, and SMLM, analysis. Functionalities: Pre = Preprocessing (Image enhancement), Pro = Processing, QA, Quantitative Analysis. year refers to release date.

Consequently, a critical gap persists between data acquisition and quantitative interpretation, as existing workflows rarely provide integrated tools for dataset comparison, multi scale blinking analysis, or generation of interpretable metrics to guide experimental decisions in real time. This limitation is particularly significant in multidisciplinary settings, where limited expertise can reduce throughput and reproducibility (Chen et al., 2024). These challenges are further amplified when working with unconventional fluorescent probes. In our study, ultrasmall core-shell silica nanoparticles (*C’dots*) with reported particle size 6.7 nm and aluminosilicate nanoparticles (*aC’dots*) of 4.8 nm exhibit distinctive blinking kinetics and high photostability, enabling STORM imaging under non-toxic conditions (Erstling et al., 2023; Erstling et al., 2021). While these properties make them attractive self-blinking platforms, driven by aluminosilicate mediated redox blinking in aC’dots, they also expose the limitations of current tools, which are not designed to robustly characterize or compare such non-standard photophysical behaviors across experimental conditions (Lee et al., 2021).

When attempting to understand both sample preparation and image acquisition in STORM, a steep learning curve becomes evident. It requires not only access to specialized microscopy systems, but also a deep understanding of sample preparation, particularly when working with non-toxic buffers or novel probes, as well as advanced computational tools for analysis. As a result, significant time must be invested in experimentation and image acquisition to understand the system under study. This complexity is further compounded by the interdependence of experimental variables such as laser power, frame rate, buffer composition, probe identity, and labeling density, which jointly influence emitter behavior and image quality. Even with experience, the iterative process of acquisition, parameter tuning, and post-acquisition analysis remains time consuming, highlighting the need for approaches that better capture population level photophysical behavior.

At a methodological level, sample preparation remains a critical yet often disconnected factor in STORM experiments. Labeling density directly impacts image quality: insufficient labeling obscures structure, while excessive labeling introduces artifacts (Elliott, 2020; Richerson et al., 2008). Despite this interdependence, current software tools treat sample preparation and imaging analysis independently, lacking integrated workflows that allow users to evaluate labeling quality prior to STORM and quantitatively assess photophysical behavior during acquisition and in a comparative methodology as well.

To address these limitations, we introduce a Python based platform that unifies quantitative analysis of sample preparation and SMLM imaging within a single user friendly framework. The approach combines confocal estimation of the *degree of labeling* (DOL), using a Stretching Open Active Contours (SOAC) framework to assess filamentous networks from single z -stacks (Li et al., 2009; Smith, 2010), with detailed extraction of STORM blinking statistics from localization data. In the filament analysis branch, rather than aiming for pixel accurate reconstruction, the software is used to extract robust geometric descriptors (e.g., continuity, length, and connectivity) that serve as proxies for labeling quality and structural organization. This integration enables a two stage optimization workflow: refining sample preparation prior to STORM and quantitatively evaluating photophysical performance during imaging.

**FIGURE 1:**
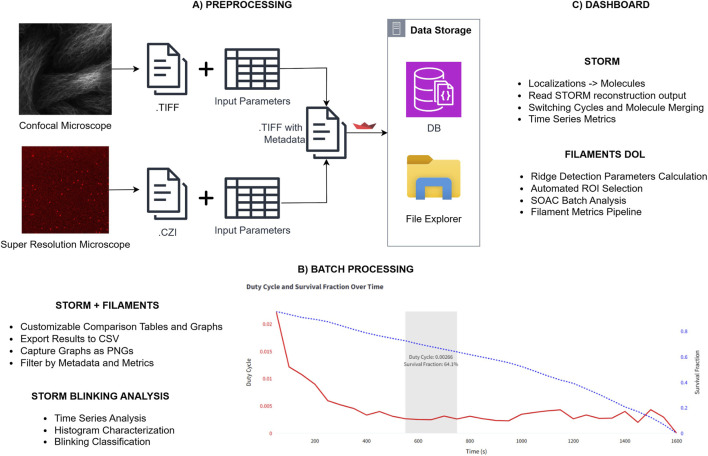
Modular analysis platform overview. The software is organized into three layers. **(A)** Preprocessing: imports raw image stacks with user defined metadata and sets up a structured folder system and database storage (DB) of hierarchical quantitative data for downstream organization. **(B)** Batch pipelines: *(1) STORM branch*: loads localization tables from ThunderSTORM or TrackMate, performs molecule merging, and computes switching cycle statistics, survival fractions, duty cycles, and other time series descriptors; *(2) Filament branch*: uses a SOAC algorithm to segment filamentous networks, extract ridge metrics, and estimate the DOL. **(C)** Interactive dashboard: provides comparative visualizations linked to experimental metadata, and supports one-click export of results. Including demonstrative graphics showcasing how time series are displayed.

Beyond individual datasets, the platform provides capabilities largely absent in existing tools, including side by side comparison, automated extraction of time-resolved photophysical metrics, and interactive visualization within a unified dashboard. In addition, it integrates a filament-based analysis that enables rapid assessment of labeling efficiency using diffraction limited imaging, allowing users to verify sample quality, monitor labeling consistency (including in live cell conditions), and identify suitable regions of interest prior to and alongside super resolution acquisition. These features support real time data quality assessment during acquisition, reducing iteration time and enabling informed experimental adjustments. Figure 1 summarizes the workflow. By combining rigorous statistical analysis with an accessible interface, the platform lowers the barrier to quantitative fluorescence microscopy while remaining flexible for advanced, user defined analysis.

This modular quantitative analysis was applied to two types of datasets: (i) confocal imaging of fluorescently labeled intracellular tubulin, and (ii) single molecule imaging of immobilized silica nanoparticles. For each pipeline, we outline the image processing steps, explain key parameter choices, and describe the statistical metrics returned to the user. We then present a comprehensive validation of both analysis modules. First, we validate the filament quantification workflow by analyzing nanobody labeled tubulin samples, demonstrating how ridge based region of interest (ROI) filtering and SOAC segmentation yield meaningful trends in filament length, intensity, contrast, and continuity across experimental conditions. Second, we benchmark the STORM pipeline with Cy5 dye to verify its ability to calculate blinking statistics that are established and validated in the literature. Finally, we report processing times and memory usage, comparing BlinkFusion’s performance against existing open-source workflows and previous manual filament analysis approaches by the group. This work demonstrates both the functionality of the app as well as its potential as a tool guiding researchers towards optimal sample preparation and image acquisition parameters for the ideal super resolution image (Erstling et al., 2021).

## 2 Methodology

The workflow applied is based on image processing and quantitative analysis towards data visualization. Noise reduction, contrast enhancement, and automated segmentation comprise the initial preprocessing stage in the quantification section (Lee and Kitaoka, 2018). In the STORM branch, sub-pixel localizations are extracted and passed to a modular pipeline that links them into molecules, computing photophysical descriptors (e.g., duty cycle, on time). In parallel, the filament based DOL branch uses ridge detection to analyze high density and low overlap regions of biopolymeric networks, which are then segmented into filaments using the SOAC framework, yielding quantitative metrics.

### 2.1 Filamentous structures analysis of DOL comparison

Evaluating DOL in diffraction limited data provides a rapid assessment of sample quality prior to specialized microscopy such as STORM, while avoiding the photophysical complexity inherent of SMLM. Beyond its role as a preparatory step, this approach also enables quantitative analysis of labeling efficiency in filamentous cellular structures using widely accessible confocal microscopy. Although confocal images reveal labeling density, they are affected by 3D crowding in the cell interior, which can bias intensity based measurements.

**FIGURE 2:**
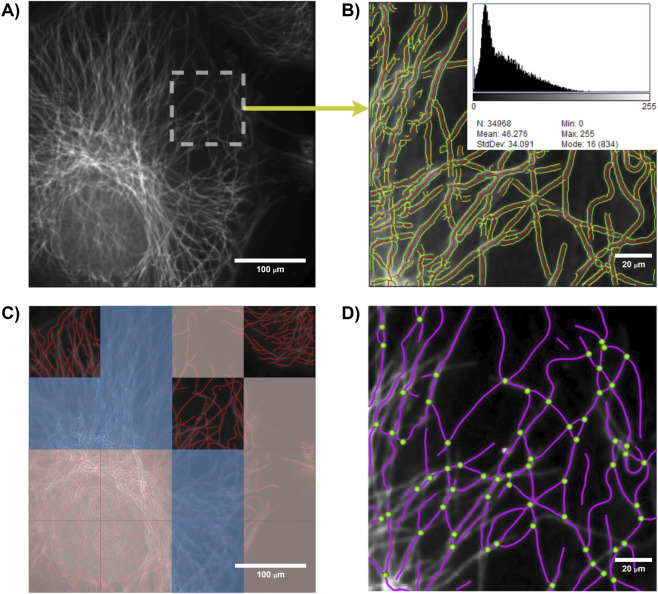
ROI selection and filament DOL analysis. **(A)** Sample: Fluorescence image of a tubulin Cy5-labeled cell showing the microtubule network. Microtubules radiate from the perinuclear Microtubule organizing center (MTOC) toward the cell periphery, where filaments are more spatially separated. **(B)** Parameter definition: a smaller ROI’s (2 × 3 section) intensity histogram (upper right) is analyzed to obtain ridge detection parameters (width, intensity contrast, mean and standard deviation of signal). Ridge detection output provides initial filament traces as shown for each region (Steger, 1998). **(C)** ROI ranking: candidate ROIs are scored by min–max filtering ( $s^{\left(1\right)}\geq 0.40$ , red tiles) and re-ranked with standardized scores (blue tiles) to identify regions with dense, well defined filaments and minimal 3D overlap (Table 2). **(D)** SOAC tracing: the selected ROIs are analyzed using Stretching Open Active Contours (SOAC), producing filament snakes and junctions for quantitative DOL and intensity analysis (Xu et al., 2015).

To address this, our grid based framework partitions images into multiple ROIs and retains only those that maximize a two-stage quality score (weights in Table 2). This strategy consistently selects peripheral regions where microtubule bundles are clearly resolved and minimally affected by axial overlap, yielding more reliable intensity measurements than densely packed central regions (See Figure 2 for a visual explanation of the process for a tubulin cell). Consequently, the estimated DOL values are more homogeneous than whole field averages (Martinez-Sanchez et al., 2013).

**TABLE 2:** ROI ranking weights and downstream filament metrics.

Metric	Min-Max Weight	Standard Weight	Description
Ridge-detection quality metrics (per ROI)			
Number of filaments	35%	35%	Count of distinct ridges; proxy for filament density
Mean length	10%	25%	Average ridge length nm
Filament junction ratio	35%	20%	Fraction of ridge pixels in junctions; network connectivity
Mean intensity	10%	10%	Average grey level intensity of ridge pixels
Coefficient of variation (width)	5%	5%	$CV=\sigma_{w}/\mu_{w}$ ; width uniformity
Coefficient of variation (length)	5%	5%	$CV=\sigma_{L}/\mu_{L}$ ; length uniformity

Metric	Formula		Description
Filament-level morphometric metrics (SOAC output, aggregated per sample)			
Contrast	$C=\frac{I_{\text{fg}}}{I_{\text{bg}}}$		Visibility of filaments over background
Signal-to-noise ratio	$\text{SNR}=\frac{I_{\text{fg}}-I_{\text{bg}}}{\sigma_{\text{bg}}}$		Signal robustness relative to background noise
Continuity (%)	$\frac{\#\left(I_{\text{fg}}>I_{\text{bg}}\right)}{\text{Total points}}\times 100$		Percentage of filament points with foreground above background
Sinuosity	$\frac{\text{Path length}}{\text{Straight distance}}$		Curvature of each filament; values >1 indicate tortuosity

Metrics are calculated for each filamentous structure segmentated by the SOAC, algorithm.

The grid based scorer thus replaces manual selection with a reproducible and scalable filtering strategy of ROIs before obtaining quantitative filaments metrics through SOAC analysis. Details on manual parameter estimation for ridge detection and batch processing, including ROI scoring and automated SOAX analysis, are provided in the Supplementary Material alongside a guide for defining the parameters for BlinkFusion (Section 5).

Tier 1: Projection, normalization, grid generation, and ridge parameterization. Multi frame TIFF stacks are averaged by z projection and normalized to 8-bit. Using the voxel size from the OME-TIFF header, we define an $N_{x}\times N_{y}$ grid of images with fixed physical pitch.

The ridge detection parameters are estimated automatically from the local image contrast of each ROI using the mean intensity and standard deviation (σ) with a Python ridge detection plugin (Wagner et al., 2017). These values, together with a user defined expected line width of ridges, determine the plugin’s Sigma, Lower_Threshold, and Upper_Threshold settings, allowing the filter to adapt to local signal variations across different ROIs without requiring manual intervention (Steger, 1998).

The batch processing computes, for each ROI, a six element descriptor vector from the ridge detection analysis, $q_{k}$ (filament count, average length, junction ratio, mean intensity, width, and length variation; see Table 2 for an example and description of parameters). This vector is used to rank ROIs.

Tier 2a: Min-max filtering. All ridge descriptors are rescaled to [0,1], and metrics that decrease with quality (width and length variation) are inverted. The resulting vector $q_{k}^{\left(1\right)}$ is projected onto the weighting vector $w^{\left(1\right)}$ to obtain a score $s_{k}^{\left(1\right)}$ . A threshold is then applied to remove (i) void tiles and (ii) regions with high junction density, typically associated with 3D crossings and inner cell structures. Accordingly, the weights are chosen to favor regions with a high number of filaments and low junction ratios.

Tier 2b: Standard-score re-ranking. The remaining tiles are standardized ( ± = 0, σ=1 ) and rescored using $w^{\left(2\right)}$ (“Standard weight” column). The top-ranked regions are then selected, either by a user-defined number or by retaining tiles with positive standardized scores. At this stage, since void and highly overlapped regions have already been removed, the scoring emphasizes ROIs with long, well defined filaments to enable more consistent comparisons across images.

The weighting parameters are determined heuristically through manual inspection across multiple datasets, adjusting weights to achieve the expected visual outcome at each filtering stage. The objective is to select regions with a high density of long, clearly resolved filaments and minimal overlap. For tubulin labeled cells, this process consistently identifies peripheral ROIs, which best represent well separated filaments due to reduced axial overlap and improved visibility at the imaging plane. As shown in Figures 2A,B, some regions may initially appear suboptimal (e.g., low intensity); however, when contrast paramaters are defined through the automated scoring process, these regions reveal well defined filaments with reduced overlap compared to more crowded areas. Notably, these peripheral regions are also commonly targeted in STORM experiments, as they better approximate ground truth for subsequent active contour fitting. As shown in Figure 2C, rejected ROIs typically correspond to low number of filaments or highly overlapped regions, while selected ROIs maximize filament separability and continuity according to the scoring metrics. A detailed guide for doing this heuristic process with the batch processing and result evaluation, is provided in the Supplementary Material (Section 5).

Tier 3: Batch SOAC and metric extraction. Each shortlisted ROI is fed to the software for quantification of biopolymer networks *SOAX*, where a parameter sweep minimizes the F -function and returns snake and junction files, see example in Figure 2D. These files are parsed into the morphometric metrics listed in Table 2, Xu et al. (2015). Since contrast, SNR, continuity, and sinuosity are computed on ROIs of matched physical extent and minimal depth artefacts, their sample level aggregates display low inter cell variance and markedly sharpen comparisons between labeling protocols, these metrics can also be useful in understanding the biological structure of cells (Hembrow et al., 2023).

In this context, the filament analysis serves both as a standalone tool for quantifying labeling efficiency in biopolymer networks and as a preprocessing step for STORM experiments. By identifying regions with optimal labeling density, continuity, and minimal overlap, it enables users to refine sample preparation and imaging parameters before super resolution acquisition. This reduces iterative trial and error before and during STORM experiments and ensures that downstream blinking analysis is performed on well prepared samples and representative regions of interest.

### 2.2 From blinking localizations to molecules in STORM

STORM imaging achieves sub-diffraction resolution by using fluorescent probes that switch between a bright “on” state and a dark “off” state. During image acquisition, only a sparse subset of molecules is photoactivated in each frame by combining high excitation power with a reducing/oxidizing (ROXS) buffer that induces reversible transitions to non-fluorescent radical or triplet states, inducing photoswitching of organic dyes (Xu et al., 2017). Activated emitters are then isolated through image processing, enabling fitting of their point spread functions (PSF), commonly using a 2D gaussian, to determine molecular positions with precisions below 200 nm. This cycle of sparse activation, widefield imaging, and precise localization is typically repeated over the entire film and the resulting coordinates are used to reconstruct the super-resolved image. Resolution scales inversely with the square root of detected photons, typically reaching ∼20 nm laterally. Critical parameters include laser power (balancing photoswitching rate and photobleaching), camera frame rate (sampling single-molecule bursts), resolution scales, and buffer composition, which together contribute for emitter density and localization precision (Rust et al., 2006).

STORM sequences may be acquired in the proprietary.czi format used by Zeiss microscopes and are automatically converted by the preprocessing module into standard.tif(f) files with embedded OME-TIFF metadata (Shroff et al., 2013; Lee et al., 2012). This ensures compatibility with downstream SMLM analysis tools (e.g., Nikon N-SIM/N-STORM) and allows the pipeline to process either native.czi or.tif(f) files without user intervention, maintaining a consistent data structure.

The TIFF stacks are saved in a user defined project folder with associated custom metadata fields (e.g., sample key, TIRF angle, laser power, buffer composition), while all acquisition metadata are stored in a MongoDB database. Localization tables are then manually generated in ImageJ/Fiji using either ThunderSTORM, which provides SMLM-specific sub-pixel fitting, or TrackMate, which enables rapid processing of large datasets but is not specifically optimized for STORM analysis (Ovesný et al., 2014; Schindelin et al., 2012; Tinevez et al., 2017). These outputs feed into the subsequent localization processing stage. A user guide for manual processing in ThunderSTORM, along with instructions for defining parameters in the STORM batch processing application, is provided in the Supplementary Material (Section 5).

Tier 1 produces a cleaned CSV of precise localization coordinates through image filtering, approximate spot detection, sub-pixel localization, duplicate removal, and drift correction in the manual ThunderSTORM process.

Tier 2 constitutes the first stage of automated batch processing, where localizations are linked into *tracks* (switching cycles) through a sequential frame procedure. At each iteration, localizations from the current frame are inserted into a KD-tree. Active tracks query this structure to identify the nearest candidate localization. Distances are evaluated both relative to the last assigned coordinate, (see Equation 1)

$$d_{\text{last}}=\sqrt{{\left(x_{\text{last}}-x_{\text{loc}}\right)}^{2}+{\left(y_{\text{last}}-y_{\text{loc}}\right)}^{2}}$$ (1)

and to the uncertainty weighted track centroid (average position see Equation 2),

$$d_{\text{weighted}}=\sqrt{{\left(x_{\text{track}}-x_{\text{loc}}\right)}^{2}+{\left(y_{\text{track}}-y_{\text{loc}}\right)}^{2}}.$$ (2)

All active tracks are compared to localizations in the subsequent frame. If the distance between a track and a candidate localization falls below a user defined threshold, the localization is assigned to the track, the gap counter is reset, and track statistics are updated. Otherwise, the gap counter is incremented; tracks exceeding the maximum allowed number of consecutive gaps are terminated. Only tracks that meet a minimum number of localizations upon termination are retained, preventing noise driven short events from being classified as valid switching cycles. Localizations in the current frame that are not assigned to existing tracks initiate new tracks. Both the maximum gap length and the minimum number of localizations are user-defined parameters.

Tier 3 aggregates tracks into *molecules*. Tracks are organized in a KD-tree, and each candidate track is compared to existing molecules by identifying the nearest centroid in a similar sequential process. Tracks are merged if their centroids fall within a user defined distance threshold (typically equal to or smaller than the localization to track threshold, given the higher precision of track centroids) and if their temporal windows do not overlap. Upon merging, track attributes such as weighted position, photon counts, and ON/OFF statistics are combined into the molecule record. Tracks that are not assigned to existing molecules initiate new molecule instances. The final output is a table of molecules with full time series attributes (ON/OFF times, duty cycle, photon yields, survival fraction), enabling downstream blinking statistics and labeling efficiency analysis.

**FIGURE 3:**
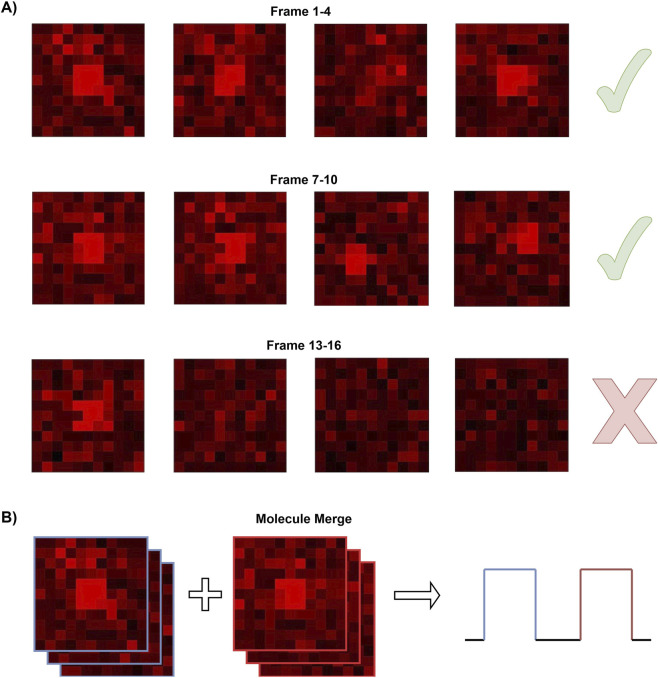
STORM processing scenario. Example of a 200×200 nm ROI illustrating how localizations are validated as tracks and merged into molecules. **(A)** *Track definition*: The first two rows show valid tracks. In the top row, consecutive frames contain PSFs that remain close in position, even when one frame is missing a localization; the maximum gap criterion allows the track to continue. In the second row, drift causes the PSF to shift laterally between frames, but because each new localization is still within the allowed distance of the track centroid, the sequence is preserved as a valid track. The third row shows an invalid case: a single PSF appears briefly but does not persist over the minimum required frames, so it is discarded as an artifact, valid in an scenario when duty cycle is longer than frame rate. (B) *Molecule definition*: Once tracks are validated, temporally non overlapping tracks with centroids within 100 nm are merged into molecules. This combines the localizations’ weighted position, cumulative photon counts, and ON/OFF statistics into a single molecular record.

This hierarchy, from localization to track to molecule, preserves spatial coherence, prevents duplicate counting, and yields a robust data structure for quantitative photophysical analysis. An example of this process is illustrated in Figure 3. Each molecule retains all ThunderSTORM reported fields (coordinates, Point Spread Function (PSF) width, intensity, background, and uncertainty; see Table 3). In the dashboard’s *Comparison* view, these metrics can be plotted between samples or analyzed individually to identify systematic shifts arising from dye selection, buffer composition, or illumination settings.

**TABLE 3:** Molecule-level comparative metrics.

Metric	Formula/Rule	Description
ThunderSTORM localization fields		
Photon counts	∑I	Total photons per localization event
Background offsets	∑offset	Integrated background offset values
Background variation	∑bkgstd	Standard deviation of background noise
Time-series metrics (tracks → molecules)		
ON times	$t_{\text{on}}={\text{End}}_{n}-{\text{Start}}_{n+1}$	Duration in frames a molecule remains continuously ON in a track
OFF times	$t_{\text{off}}={\text{Start}}_{n}-{\text{End}}_{n-1}$	Duration of dark intervals between ON states
Switching cycles	Num Tracks	Total number of ON/OFF cycles per molecule
Duty cycles	$DC=\frac{\sum t_{\text{on}}}{T_{\text{acq}}}$	Fraction of acquisition time spent in the ON state
Survival fractions	$SF\left(t\right)=\frac{N_{\text{active}}\left(t\right)}{N_{\text{total}}}$	Proportion of molecules still active at time t
Photon yields	∑Photons	Total photons emitted during ON states
Single-molecule blinking classifications		
Blinks on once	DC≤0.5 , cycles =1	Single ON event followed by permanent OFF state
Blinks off once	DC>0.5 , cycles =1	Mostly ON with a single OFF transition
Blinks on multiple	DC≤0.25 , cycles >1	Predominantly OFF with frequent ON events
Blinks off multiple	DC>0.25 , cycles >1	Predominantly ON with frequent OFF interruptions
Photobleach	End Frame<Final Frame	Emission terminates before end of acquisition
Inverse Photobleach	End Frame=Final Frame	Emission persists until final frame
Always ON	No OFF transitions	Continuous emission throughout the experiment

ThunderSTORM, metrics are weighted for dashboard comparison. Time-series and QE, metrics are computed over non overlapping 50 s windows, with averages weighted by the number of tracks per molecule to favor well sampled emitters.

Beyond localization metrics relevant to detection accuracy, we compute a second tier of *time series metrics* following methods established by Dempsey et al. (2011). Each molecule’s switching cycles are grouped into consecutive, user defined time windows (in seconds), within which weighted averages of ON-time, OFF-time, duty cycle (DC), cycle count, photon yield, and survival fraction are calculated. We then identify a *quasi-equilibrium* (QE) period, ${\bar{DC}}_{\text{QE}}$ , defined as the interval with minimal DC standard deviation $\left(\sigma_{\min}\right)$ : (see Equations 3, 4)

$$\sigma_{\min}=\sqrt{\frac{1}{n}\overset{n}{\underset{i=1}{\sum }}{\left({DC}_{i}-\bar{DC}\right)}^{2}},\;n=5$$ (3)

$${\bar{DC}}_{\text{QE}}=\frac{1}{n}\overset{n}{\underset{i=1}{\sum }}{DC}_{i}$$ (4)

The QE window yields a stable duty cycle indicator. Users can compare whole population versus QE restricted metrics via histograms, scatter plots, and statistical tests.

Finally, each molecule is also assigned a *blinking class* (e.g., “Blinks on once,” “Always ON,” “Photobleach”) using the rules in Table 3, adapted from Binkley and Griffin, to enable rapid screening of blinking profiled subpopulations (Binkley and Griffin, 2021).

## 3 Results

BlinkFusion provides an open source intuitive, end to end interface; it will be maintained over time, with upcoming support for 2D and 3D imaging. An App usage guide is included in the Supplementary Material 5. During preprocessing, users drag files or folders into the app, which parses Zeiss Elyra CZI/TIF or Andor/Olympus spinning-disk TIFF stacks to extract metadata (pixel size, laser wavelength, acquisition date). Any missing information such as sample type, dye concentration, and buffer is entered via an auto suggest form that enforces consistent terminology. Once complete, images are organized into a user defined folder hierarchy, and for STORM datasets all entries are stored in MongoDB.

In the processing stage, the workflow splits into two pipelines. For filament analysis, ridge detection, SOAC segmentation, and ridge metric extraction can be run immediately after preprocessing. For STORM, emitters are first manually obtained in ThunderSTORM via Fiji; the app then can automatically assemble tracks (switching cycles), merge molecules, and compute metrics described earlier. Unprocessed images are flagged, allowing a single “batch” command to analyze multiple files unattended, while still permitting per file parameter adjustments if needed.

The dashboard integrates global comparison with detailed inspection. A metadata filter restricts data by microscope, date, sample, etc., essential for exploring large experiments. Filtered datasets appear in a comparative table and can be plotted as line, bar, box, or violin plots. To capture STORM’s stochasticity, the dashboard also offers per-image analytics: time-series curves (duty cycle, survival fraction), histograms of ON-time and cycle counts with an outlier slider, and a single molecule trace viewer showing coordinates, photon counts, and blinking class labels. A toggle confines plots to the quasi-equilibrium window for benchmarking across movies. All graphics export as PNG and tables as CSV for external analysis and presentation.

### 3.1 Filaments tool validation

The filament workflow uses batch processing of established methods, ridge detection for ROI selection and SOAX for segmentation, already validated in the literature (Steger, 1998; Xu et al., 2015). Our validation focused on two questions: (i) does the weighted ROI filter enable fair comparisons across samples, and (ii) do the derived metrics (length, intensity, contrast, continuity) correspond to changes in labeling or imaging. Filter weights were initially tuned manually by reviewing hundreds of candidate ROIs until selected regions consistently showed clear, well separated filaments.

#### 3.1.1 Sample preparation and imaging

Tubulin was selected as the test structure because its well characterized cytoskeletal architecture provides a dense yet spatially organized filament network, making it an ideal benchmark for evaluating filament continuity and labeling efficiency (Schvartz et al., 2017). Unlike more heterogeneous or sparse cellular structures, microtubules form extended, quasi-linear filaments that are highly sensitive to variations in labeling density and imaging conditions, directly impacting continuity based metrics. This makes them particularly suitable for validating ridge detection and SOAC based segmentation approaches. Additionally, tubulin is a relevant biological target in the context of aC’dots, as these probes are designed for live cell imaging applications where cytoskeletal structures such as microtubules are commonly studied (Erstling et al., 2021).

Cells were fixed with paraformaldehyde, permebealized and blocked following the UCLA Brain Research Institute immunofluorescence protocol, then immunostained with nanobody conjugated aC’dots. Both direct and indirect labeling routes were explored, but the indirect method, primary antibody followed by fluorescent secondary, proved advantageous for signal amplification and multiplexing potential (Im et al., 2019). To ensure reproducibility, labeling density was carefully monitored, since uneven staining could bias filament continuity metrics.

A total of 39 cell images were acquired, with 13 images per aC’dots concentration (10, 60, and 80 μ g ${\text{ml}}^{-1}$ ), across three independent days and samples. Imaging was performed using a spinning disk confocal microscope (Andor/Olympus) at 512 × 512 pixels, with a pixel size of 0.80 μ m, laser power of 10 mW, and 25 Z-frames per field (Graf et al., 2005). Z-stacks were averaged to maximize signal to noise ratio (SNR), yielding a 2D projection comparable to datasets used for STORM analysis. Additionally, three datasets for the 10 and 60 μ g ${\text{ml}}^{-1}$ concentrations were acquired at an increased laser power of 20 mW to assess the effect of excitation intensity as a secondary variable, while maintaining a comparable average filament density. The parameters values for ridge detection analysis and SOAX are presented in the Supplementary Material (Section 5).

To standardize the analysis, each projection was divided into a grid of 16 candidate ROIs (32 × 32 pixels each). These tiles were automatically ranked based on filament quality metrics, including ridge density and mean filament length. The weighted filtering values are defined in Table 2. During the min–max filtering stage, a conservative threshold of $s_{k}^{\left(1\right)}\geq 0.40$ was applied. Because the *number of filaments* and *junction ratio* together account for 70% of $w^{\left(1\right)}$ , this stage effectively acts as a binary filter, removing both low filament regions (background) and highly dense, overlapping regions, thereby preferentially selecting peripheral regions of the cell.

For the standardized scores, an additional 15% weight is assigned to *mean length*, favoring long, continuous filaments while de-emphasizing residual overlaps by reducing the weight of the filament junction ratio, as the min-max filtering step is expected to have already removed inner cell regions. The top three scoring ROIs per cell, typically located near the cell periphery (Example in Figure 2), were selected and passed to SOAX, resulting in 2-3 ROIs per image after filtering.

**FIGURE 4:**
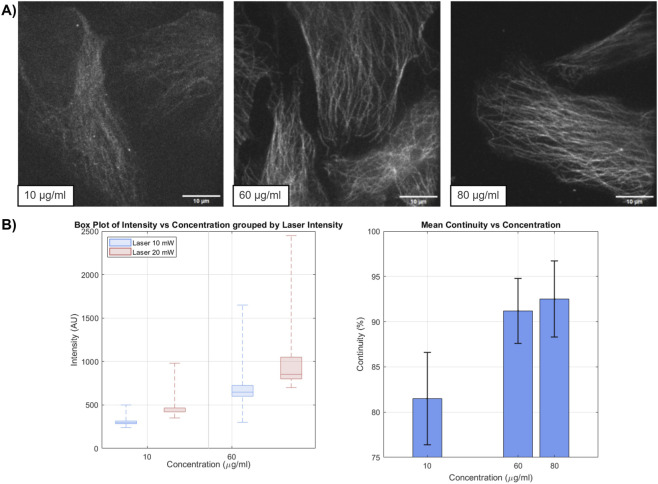
Filament imaging and quantitative metrics for Nanobody-aC’dots. **(A)** Representative spinning-disk confocal images of tubulin labelled with Nanobody-aC’dots at 10, 60 and 80 μ g ${\text{ml}}^{-1}$ (left to right). Low concentration yields sparse, poorly defined filaments, whereas 60 μ g ${\text{ml}}^{-1}$ already produces continuous networks; further increase to 80 μ g ${\text{ml}}^{-1}$ offers no obvious improvement. Scale bars: 10 μ m. **(B)** left: Box plot of mean filament intensity (arbitrary units) as a function of concentration, color grouped by laser power. Both higher dye loading and stronger illumination enhance photon output, reflected in the upward shift of the intensity distribution. **(B)** right: Bar plot of mean filament continuity versus concentration for Laser 10 mW. Continuity rises sharply between 10 and 60 μ g ${\text{ml}}^{-1}$ and plateaus thereafter, in agreement with the qualitative observations in panel **(A)**.

These ROIs were then processed using SOAX segmentation, where SOACs analysis was applied to extract filament centerlines and junctions. This pipeline reduces subjective bias and ensures that the selected ROIs correspond to peripheral regions typically used in STORM imaging. For the analysis reported in Figure 4 and Table 4, an average of 165 filaments per image was detected, with a minimum of 62 and a maximum of 217 filaments. This corresponds to approximately 2000 filaments analyzed per concentration.

**TABLE 4:** Filament metrics for Nanobody-aC’dots for varying dye concentration and laser power.

µgml^-1^	mW	Length (µm)	Intensity (a.u.)	Contrast	Continuity (%)
10	10	2.898±0.65	296±24	1.098±0.053	81.12±8.03
10	20	3.025±0.87	423±55	1.120±0.061	82.59±7.82
60	10	3.455±1.04	648±101	1.303±0.138	91.54±6.94
60	20	3.730±1.19	1058±242	1.365±0.159	92.82±5.55
80	10	3.798±1.28	852±212	1.389±0.19	93.52±5.66

Metrics include filament length, intensity, contrast, and continuity. Higher concentrations and laser intensities correlate with improved filament quality and continuity.

#### 3.1.2 Qualitative observations

Figure 4A shows representative images at 10, 60 and 80 μ g ${\text{ml}}^{-1}$ dye concentration. The jump from 10 to 60 μ g ${\text{ml}}^{-1}$ markedly improves filament definition, whereas a further increase to 80 μ g ${\text{ml}}^{-1}$ offers little additional benefit, an impression corroborated by the quantitative results.

#### 3.1.3 Quantitative metrics

Box-plot analysis in Figure 4B confirms that filament intensity rises with both concentration and laser power, yet the simultaneous growth in contrast demonstrates that the brightness gain is not merely background amplification. Continuity, plotted in Figure 4B right, climbs from 81% at 10 μ g ${\text{ml}}^{-1}$ to >93% at 80 μ g ${\text{ml}}^{-1}$ , indicating a near complete ridge reconstruction. Table 4 condenses these findings across all five acquisition settings: higher dye loading and laser power correlate with longer filaments (3.80μm) , brighter signals (up to 1058 au) and superior continuity.

#### 3.1.4 Interpretation and outlook

The monotonic improvement of every metric with concentration and laser power validates the analysis pipeline to the initial assessment of the images with varied concentration: each quantity scales in the physically expected direction, and the plateau from 60 to 80 μ g ${\text{ml}}^{-1}$ highlights a practical upper limit before phototoxicity or dye cost become prohibitive. As with the STORM module, these results can be explored interactively in the dashboard: global filters isolate specific concentrations or laser powers, side-by-side plots reveal trends. The ability to apply the same comparative toolkit to both STORM and filament data for comparative analysis underlines the generality of the application and completes the validation of its dual analysis design.

### 3.2 STORM tool validation

Reliable benchmarking is essential to show our STORM dashboard reproduces accepted blinking statistics. As a first sanity check for datasets analyzed, we compared (i) merged localizations per switching cycle obtained from the batch processing and (ii) raw switching cycle counts to ThunderSTORM’s “merge tracks” output through post processing options, finding a relative error of approximately 10% due to our additional cross-checks (Ovesný et al., 2014). While this agreement is encouraging, it does not validate the higher order time series metrics (duty cycle, survival fraction, photon yield) that underpin population level analysis.

To address this, we used the benchmark dataset from (Dempsey et al., 2011)^1^. We selected Cyanine 5 (Cy5) as the sample examined, since this is an extensively used dye in the super resolution studies and has been previously used for fluorescent dye with C’dots and aC’dots (Erstling et al., 2021; Herz et al., 2009; Gopika et al., 2021). We then extracted three key photostatistics from each movie and compared them to Dempsey’s reported values:

1. The average duty cycle per molecule in a QE interval.
2. The survival fraction after 400 s (matching the 400–600 s window in the literature).
3. The mean photon count per switching cycle.

### 3.2.1 Sample preparation and image processing

Samples were prepared by immobilizing Cy5 particles onto glass surfaces for single particle imaging, following previously published protocols (Kohle et al., 2019). Surface functionalization with silanes and streptavidin enabled sparse immobilization of biotinylated particles, minimizing clustering and ensuring reliable capture of single emitter kinetics. Imaging buffers were prepared according to established formulations to ensure consistent photoswitching conditions (Erstling et al., 2021; Syga et al., 2018), incorporating thiols and an enzymatic oxygen scavenger system to enhance fluorophore stability and switching contrast.

Movies were acquired using a Zeiss Elyra super resolution microscope (BmE, 647 nm, ∼2 kWcm^-2^ at the sample). Each acquisition consisted of 40,000 frames at 100 × 100 pixels, with a pixel size of 100 nm and an exposure time of 30 ms, resulting in total acquisition times of approximately 20 min. The camera gain was set to 100, using a Laser HR Diode 642–150. Parameters used for ThunderSTORM and subsequent batch processing are detailed in the Supplementary Material (Section 5).

Each frame was processed in ThunderSTORM using a wavelet filter and Gaussian smoothing for sub-pixel localization, while omitting the “seed in frame 0” step (Shimizu, 2007).^2^ This modification reduces bias introduced by the high emitter density in initial frames, improving the accuracy of downstream quantitative analysis.

In contrast to earlier workflows, which discard emitters within five pixels (800 nm) of a neighbor in the first frame to avoid PSF overlap, our approach relies on per-frame detection combined with sub-pixel Gaussian fitting, eliminating the need for this initial filtering step (Dempsey et al., 2011). Drift correction was additionally applied post-acquisition to account for nanoscale sample motion. Together, these refinements produce cleaner trajectories and preserve localizations that would otherwise be excluded, which is particularly important for dense cytoskeletal samples.

Localizations originating from the same emitter were linked into tracks (switching cycles), allowing up to 2 consecutive missed frames and applying a 200 nm distance threshold. Tracks were required to contain at least 3 localizations to be retained. Completed switching cycles were subsequently merged into molecules using a stricter 100 nm criterion (see methodology in Section 2.2). This hierarchical grouping enables robust extraction of molecular descriptors, including ON/OFF times, duty cycle, and survival fraction. To enable direct comparison with the time-series analysis reported by Dempsey et al. (2011), a temporal binning interval of 50 s was used.

### 3.2.2 Interpretation and outlook

**FIGURE 5:**
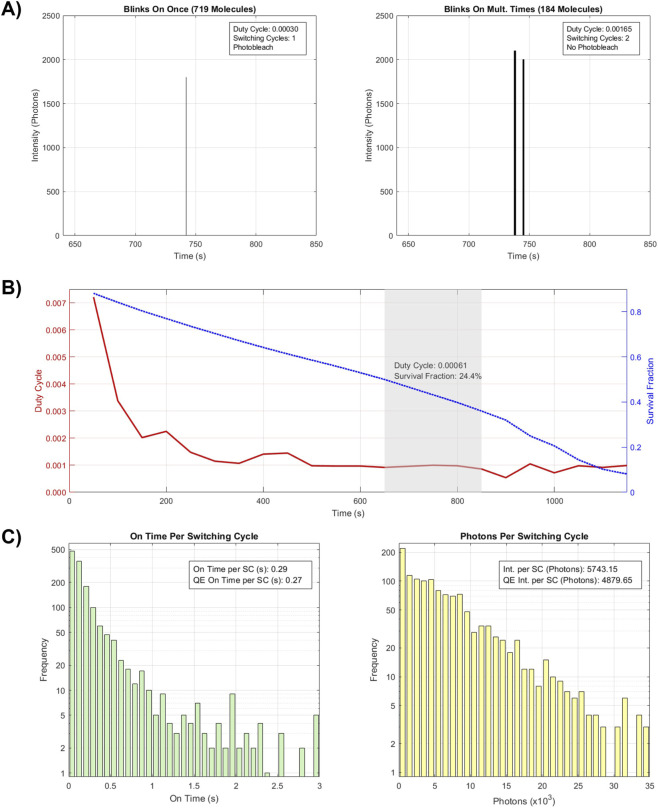
Cy5 blinking statistics characterization. Statistics are correspondent to the results exported for one of the Cy5 datasets averaged in Table 5. **(A)** Intensity traces in the QE window for single blink (left, 719 molecules) and multi blink (right, 184 molecules) emitters. The dashboard automatically splits these sub-populations and overlays their photon statistics. **(B)** Duty cycle (red) and survival fraction (blue, right axis) time series. Both curves decay rapidly after laser activation and plateau in the QE band (grey). Global DC and SF values at the QE midpoint are annotated. **(C)** Histograms of ON times (left) and photon yield per switching cycle (right) within QE. Insets compare whole trace means with QE restricted means, underscoring the stability of the selected interval. Together, panels (b–d) demonstrate how the application isolates the stationary regime and outputs per-molecule statistics that can be compared directly to the Cy5 benchmark.

From the results obtained in Table 5, the key photophysical metrics show good agreement with previously reported values for Cy5 dyes (Dempsey et al., 2011), with relative errors generally within ∼20%. A comparable number of active molecules were analyzed in both studies, where the active population is defined as emitters that remain fluorescent during the quasi-equilibrium (QE) period without photobleaching. In particular, the duty cycle (DC), one of the most critical parameters for assessing labeling performance in SMLM, remains within the same order of magnitude as the reference. Similarly, survival fraction and photon yield per cycle show modest deviations, indicating that the pipeline accurately captures the essential blinking behavior of Cy5 despite differences in the localization and tracking workflow. These results support the validity of the automated analysis framework and its ability to reproduce established benchmarks while reducing manual intervention. An example Figure 5 shows the detailed blinking statistics for one of the datasets analyzed.

**TABLE 5:** Cy5 single-molecule metrics: present study vs. literature.

Metric	This work(Avg ± SD, n=2 )	Dempsey’12	Rel. error (%)
Duty cycle, DC	$\left(8.3\pm 0.9\right)\times 10^{-4}$	$7.0\times 10^{-4}$	+18.6
Survival fraction, SF (%)	73.5±9.9	83	−11.4
Photon yield per cycle	5 103±267	5873	−13.1
Switching cycles, SC	1.51±0.21	17	−91.1
Active population, AP	3 032	3000–4000	NA

Values from this work are the mean of two Cy5 data sets; literature numbers are taken from the summary table in Dempsey et al. DC, SF, and photon yield show discrepancies below $\tilde{2}0\%$ , confirming that the dashboard reproduces benchmark behaviour despite the stochastic nature of STORM, imaging.

Previous studies typically report the total number of switching cycles accumulated over the full acquisition and subsequently identify a quasi-equilibrium (QE) interval within that time range. In contrast, our approach quantifies switching cycles exclusively within the QE window, rather than across the entire movie. This distinction is important, as it removes the dependence of the metric on acquisition length and frame rate, enabling more consistent comparisons across experiments and imaging conditions. It is particularly advantageous for smaller datasets, where metrics derived from full length acquisitions can be biased by limited sampling.

Restricting the analysis to the QE regime also reduces two major sources of bias: (i) the initial high density phase, where overlapping emitters and rapid photobleaching can artificially increase apparent switching activity, and (ii) long acquisitions, where extended imaging of non-photobleached emitters can accumulate additional switching events over time, artificially inflating cycle counts. Consequently, the number of switching cycles reported here is lower than values obtained from full trace analyses, but more accurately reflects steady state fluorophore behavior under stable imaging conditions.

The STORM dashboard reproduces the benchmark photophysics of Cy5 with quantitative fidelity while keeping the analysis fully transparent: every localization is filtered, linked and visualized on a frame by frame basis. Because the workflow is parameter driven rather than dye specific, the same pipeline transfers directly to other fluorophores and imaging conditions. Figure 5 assembles the central read-outs, reconstruction, per molecule traces, global time-series and QE histograms, into a single view that showcases this end to end capability.

### 3.3 Blinkfusion’s processing efficiency

For the processing efficiency analysis, a comparison was made between the process conducted using Fiji/ImageJ and those performed directly in the BlinkFusion app. The metrics were gathered on a laptop with an Intel Core i7-8750H CPU (6 cores, 12 threads), 16 GB of RAM (2667 MHz and 3200 MHz), and storage consisting of a 512 GB ADATA SSD and a 1 TB ST1000LM035-1RK172 HDD, running Windows 10 Home, version 10.0.19045.

**FIGURE 6:**
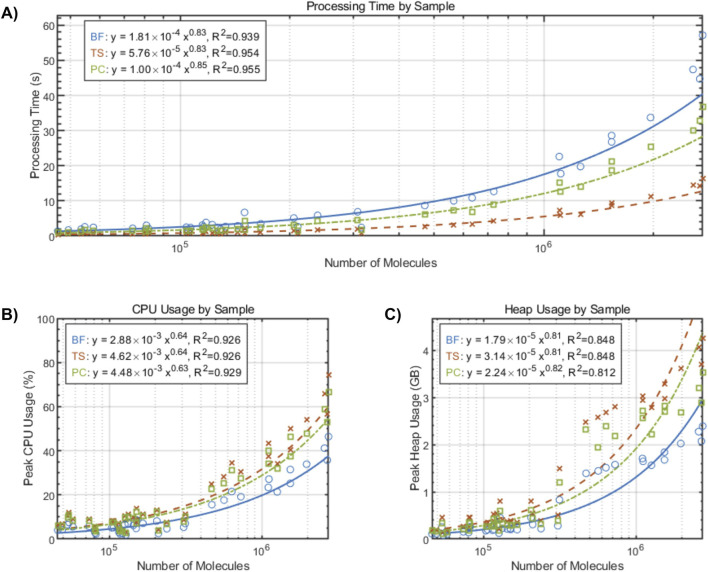
Processing efficiency metrics for STORM workflow. TS denotes *ThunderSTORM*, BF refers to *BlinkFusion*, and PC denotes the *Picasso* framework. The horizontal axis indicates the number of localizations. **(A)** Processing time (seconds) required for the merging step as a function of localization count. **(B)** Peak CPU usage, measured as the maximum percentage of total logical cores utilized. **(C)** Peak heap memory usage, defined as the largest resident heap allocation during processing. ThunderSTORM achieves the fastest runtimes due to its simplified merging strategy. BlinkFusion, implemented in Python with additional processing steps, trades speed for reduced CPU and memory usage. Picasso shows intermediate behavior in all three metrics, reflecting a balance between computational efficiency and algorithmic complexity.

To assess STORM processing performance, we compared three representative tools: ThunderSTORM (TS), BlinkFusion (BF), and Picasso across 40 datasets spanning a wide range of localization counts (Figure 6). ThunderSTORM was selected as a baseline due to its widespread adoption in the field, while Picasso was included as a complementary tool which also provides quantitative analysis with closest approach to our application without including time series or comparative analysis. ThunderSTORM and Picasso applies a basic track merging strategy without weighted localization or proximity based merging of tracks, allowing for faster computation as dataset size grows. In contrast, BlinkFusion incorporates these additional checks, which increase its computational load. We emphasize that this comparison is limited to processing time, CPU usage, and memory consumption, as these are relevant metrics consistently available across all tools. The time series and comparative metrics introduced in BlinkFusion are not implemented in existing software, and therefore cannot be directly benchmarked.

As shown in Figure 6A, processing time scales with localization count following a power law trend. ThunderSTORM achieves the fastest runtimes due to its simpler merging strategy, while BlinkFusion exhibits slower scaling due to additional operations such as weighted localization and proximity based track merging. Picasso shows intermediate performance between both methods. BlinkFusion also displays increased variability for larger datasets, likely due to adaptive heuristics and memory-intensive processing steps.

In terms of resource usage (Figures 6B,C), BlinkFusion consistently requires less CPU and memory than ThunderSTORM, while Picasso narrows on the processing required by ThunderSTORM. These results highlight a trade off between speed and computational efficiency: ThunderSTORM prioritizes runtime, whereas BlinkFusion reduces resource consumption and enables extended analytical capabilities. Picasso provides a balance between both approaches but lacks support for comparative and time series analysis.

For filament DOL analysis, we processed 50 images, omitting scatter histogram plots because frame and filament counts varied minimally. We first replicated our manual workflow, measuring only the initial ridge detection step, excluding SOAC segmentation to isolate ROI selection time. This manual approach incurs an extra 30 s for software switching and must be repeated three times to obtain the top three ROIs. In contrast, BlinkFusion performs identical ridge detection and top-3 ROI selection in a single pass (Section 2.1), delivering a 76% reduction in runtime (Table 6).

**TABLE 6:** Comparison of processing metrics for filament DOL.

Method	Time (s)	Peak CPU (%)	Peak heap (MB)
Manual	39.6 ± 3.2	18 ± 3.2	1567 ± 143
BlinkFusion	9.5 ± 2.1	21 ± 2.8	1724 ± 178

Metrics shown reflect average processing time, peak CPU, usage, and peak heap memory across test images for manual and BlinkFusion methods.

Despite this speedup, BlinkFusion’s peak CPU and heap usage rise by only 17% and 10%, respectively, demonstrating efficient in-memory buffering and minimal per-ROI overhead. The unified loop avoids repeated plugin loads, yielding sub-linear scaling with ROI count. Parallelizing ROI analysis could further reduce total processing time at the cost of a proportional increase in aggregate CPU load and memory making BlinkFusion ideal for high throughput applications under resource constraints.

## 4 Discussion

BlinkFusion is a modular platform that unifies two previously separate workflows, single molecule blinking analysis and filament DOL quantification, within a single interface. By organizing datasets, tracking metadata, and running batch pipelines, it lets users move directly from acquisition to side by side views of filament structure and blinking behavior without switching software or writing scripts. Experiments can be configured, run, and rerun in real time through the app.

Validation on representative datasets confirmed the accuracy of both analysis branches. The filament pipeline, evaluated using nanobody labeled tubulin, showed that intensity, contrast, and filament continuity follow the expected dependence on dye concentration and illumination, with a plateau observed between 60 and 80 μ g ${\text{ml}}^{-1}$ . These trends were robustly captured through the combination of weighted ROI filtering and SOAC-based segmentation, demonstrating that structural and photophysical information can be jointly extracted and consistently interpreted.

For Cy5, the STORM pipeline reproduced duty cycle, survival fraction, and photon yield within 20% of reported literature values. The slightly reduced number of switching cycles is attributed to a stricter quasi-equilibrium window, which minimizes early frame overlap and drift artifacts. Importantly, the integration of filament analysis with STORM processing proved critical for understanding labeling efficiency in aC’dots and for optimizing imaging conditions, resulting in improved image quality and more consistent quantitative metrics, including lower duty cycle and higher photon yield. Additional analysis on C’dots and aC’dots further support the utility of this framework for improving sample preparation and acquisition strategies while providing an initial input into these particles blinking statistics (Salgado, 2024).

BlinkFusion’s batch engine can process millions of localizations in minutes, and ridge detection with SOAC fitting replaces manual Fiji workflows. All functions are accessed through a user friendly graphical interface, so both microscopy specialists and interdisciplinary teams can obtain quantitative answers without coding. Although the localization merging algorithm sacrifices some raw speed, it gains accuracy and consistency while reducing CPU and memory load, advantages for large or hardware limited studies. Meanwhile, the filament DOL analysis makes this type of analysis accessible, by reducing processing time while maintaining similar CPU and memory load. In both cases we see that the possibility of optimization is possibly accessible through parallelization and usage of more effective data structure. Integrated ROI scoring and resource aware execution make the platform suitable for high throughput environments.

While the tool was designed with biological applications in mind, its modular design and general purpose metrics make it applicable beyond cell imaging. The filament pipeline detects and scores curvilinear features independently of sample type as shown in Table 4. With the expansion of fluorescence microscopy and super resolution microscopy from live cells to other fields such as inline circuit design defect detection, this tool promises to be a valuable resource for researchers to analyze labeling in multiple applications, as well as improve the quality of their super resolution images by better understanding the blinking statistics of their samples and offering tools to improve them in real time (Nguyen et al., 2022; Yang et al., 2021; Nature Methods, 2015).

Still, some limitations remain. Initial localization of emitters must currently be performed in ThunderSTORM, and tighter integration of that step would make the pipeline fully self contained. Also, the ROI ranking in the filament module was tuned through scaling parameters of images; a more general scoring system, optimized using machine learning or Bayesian parameter selection, could make the tool more robust across sample types and imaging platforms. Finally, the app currently only supports 2D datasets for both STORM and filament analysis; in future iterations, this will be expanded to include 3D analysis.

Overall, BlinkFusion links blinking analysis and filament quantification in a way that helps users optimize experiments in real time. By adjusting sample preparation or acquisition settings and reprocessing with just a few clicks, users can evaluate the impact of changes on both structural and photophysical readouts. We see this as an important step toward a more iterative, intuitive, and automated way of doing fluorescence imaging, one where the software provides immediate, quantitative feedback and where pipelines can be extended or adapted to new tasks as they emerge.

## 5 Conclusion

BlinkFusion is a modular, open source platform that unifies blinking statistics with filament labeling efficiency into a single, reproducible workflow, providing immediate quantitative feedback for optimizing fluorescence microscopy experiments. By correlating photophysical dynamics with structural quantification, the platform facilitates real time adjustments to sample preparation and acquisition parameters, with subsequent reprocessing enabling precise evaluation of their effects. Validated on representative STORM and confocal datasets, BlinkFusion supports robust benchmarking of dyes, buffers, and imaging conditions with reduced manual effort. While future extensions toward 3D data and tighter integration with localization tools will broaden its scope, BlinkFusion already offers a practical and accessible solution for quantitative fluorescence analysis.

## Data Availability

The datasets presented in this study can be found in online repositories. The names of the repository/repositories and accession number(s) can be found below: https://dx.doi.org/10.21227/ytqw-t131.

## Footnotes

1 The “quasi-equilibrium” (QE) interval as defined by Dempsey et al. is the period of minimal duty cycle variance for molecules which have not photobleached until a certain period of time, indicating photophysical stationarity.

2 In Dempsey’s workflow, molecules are first detected in frame 0 by thresholding pixels exceeding four times the background standard deviation, followed by removal of detections within five pixels (160 nm) and signal integration over a 7 × 7 window for subsequent frames (Dempsey et al., 2011).
