# Supplementary material for: BlinkFusion: modular platform quantifying labeling efficiency and photophysics in regular and super-resolution fluorescence microscopy
Source: Front Bioinform. 2026 May 28;6:1817759. doi: 10.3389/fbinf.2026.1817759 (PMC13253799; doi:10.3389/fbinf.2026.1817759)
Supplement: Supplementary file 1 [file DataSheet1.pdf]

Supplementary Material: BlinkFusion

In this supplementary material, we provide the BlinkFusion usage guide, an explanation of the use of ridge detection software and parameter selection for our specific use case, the ThunderSTORM preprocessing workflow required prior to STORM analysis, and a definition of the Filament and STORM validation parameters used during the validation and results section.

1 SOFTWARE VALIDATION PARAMETERS

For the datasets analyzed for both Filament Analysis and STORM processing, we are including here the parameters selected for processing in each of the steps of the application usage for reproducibility and transparency purposes.

1.1 Filaments Processing Parameters

For the filament analysis, three main groups of parameters are required: (i) ridge detection parameters (when manually defined), (ii) region of interest (ROI) selection parameters, including weights and number of regions analyzed, and (iii) parameters for the SOAX software.

The ridge detection parameters Wagner et al. (2017) were computed automatically for each ROI as described in Section 3. The line width was manually set based on an average of 10 pixels (corresponding to 0.8 μm per pixel), while the high and low contrast thresholds were estimated automatically. This configuration allows the filter to adapt to local variations in intensity and contrast across the image.

The parameters used for weighted ROI selection were determined through a manual calibration process across multiple images. This process consisted of identifying which combinations of metrics most consistently enabled: (i) the exclusion of regions with little or no filament content, and (ii) the removal of highly dense regions, such as the interior of the cell, where filament overlap is significant. For the final selection stage, weights were further refined to prioritize ROIs located near cell edges, which typically provide clearer and more comparable filament structures. Additional details on this procedure are provided in Section 3.2, and a more detailed discussion of the weighting strategy is included in the main manuscript.

Finally, the parameters for the Stretching Open Active Contours (SOAC) software were set to the default values provided by the implementation, as recommended in the original work Xu et al. (2015). Although these parameters can be tuned, SOAX incorporates an internal optimization process that adapts to the specific characteristics of each image. Therefore, using consistent initial values while allowing the algorithm to converge during optimization is appropriate when analyzing datasets with varying intensity profiles. The default parameter values used for all datasets are listed in Table S1. For a detailed description of each parameter, we refer the reader to the SOAX user guide Xu et al. (2015).

1.2 STORM Processing Parameters

For the STORM processing step, a manual stage is required for localization detection prior to obtaining quantitative metrics. For a detailed description of the workflow, please refer to Section 4. The parameters used for the STORM validation datasets are summarized in Table S2.

Wavelet based filtering methods, often combined with band-pass approaches, are widely used due to their robustness in threshold determination Shimizu (2007). The default thresholding strategy in ThunderSTORM,

Parameter	Value
intensity-scaling	0
gaussian-std	0
ridge-threshold	0.01
maximum-foreground	65535
minimum-foreground	0
init-z	true
snake-point-spacing	1
minimum-snake-length	10
maximum-iterations	10000
change-threshold	0.1
check-period	100
alpha	0.01
beta	0.1
gamma	2
external-factor	1
stretch-factor	0.2
number-of-background-radial-sectors	8
background-z-xy-ratio	2.88
radial-near	4
radial-far	8
delta	4
overlap-threshold	1
grouping-distance-threshold	4
grouping-delta	8
minimum-angle-for-soac-linking	2.1
damp-z	false

Table S1. SOAX processing parameters used for filament analysis.

Category	Parameter	Value
Image filtering	Filter	Wavelet filter (B-Spline)
	B-Spline order	3
	B-Spline scale	2
Approx. localization	Method	Local maximum
	Peak intensity threshold	std(Wave.F1)
	Connectivity	8-neighbourhood
Sub-pixel localization	Method	PSF: Gaussian
	Fitting radius [px]	3
	Fitting method	Maximum likelihood
	Initial sigma [px]	1.6
	Multi-emitter fitting	Disabled
Intensity filtering	Limit intensity range	Disabled
	Intensity range [photons]	NA
	Same intensity for all molecules	NA
Visualization	Method	Normalized Gaussian
	Magnification	50
	Update frequency [frames]	50

Table S2. ThunderSTORM processing parameters for STORM validation.

based on the standard deviation of the wavelet decomposition, typically provides reliable results. Molecule detection is performed using a local maximum approach, which is one of the most commonly used methods in single molecule localization microscopy (SMLM) algorithms Křížek et al. (2011).

After thresholding and estimating approximate emitter positions, ThunderSTORM refines localizations using a Gaussian point spread function (PSF) model. The fitting radius was set to 3 pixels, considering a pixel size of 100 nm, and an initial sigma of 1.6 was used. This configuration assumes that the initial sub-pixel localization falls within the range of approximately 200-400 nm, consistent with the diffraction limit. The final localization is obtained through the fitting process within this neighborhood. Additional details on parameter selection can be found in the ThunderSTORM documentation Ovesný et al. (2014).

Following localization, two additional processing steps were applied. First, duplicate localizations were removed using a distance threshold of 200 nm. For this analysis, the goal is not to capture the entire localization population, but rather to characterize blinking behavior without overfitting; therefore, duplicate removal serves as a filtering step. Second, drift correction was applied using the cross-correlation method, which aligns spatial patterns across consecutive frames Křížek et al. (2011); Ovesný et al. (2014). The resulting images were then processed directly in the application.

Parameter	Value
min_frames_locs_to_tracks	3
max_gaps_locs_to_tracks	2
max_distance_merge_localizations	200 nm
max_distance_merge_molecules	100 nm
time_series_interval_seconds	50 s

Table S3. STORM batch processing parameters used for tracking and time-series analysis.

Finally, the parameters for batch processing, in Table S3 are defined as described in Section 2.1.2. The minimum number of frames was set to 3, corresponding to a minimum on-time of approximately 0.9 s. This choice was motivated by the observation that Cy5 fluorophores in our samples exhibit an average on-time of approximately 0.20 s, while also presenting occasional high-intensity bursts that should not be considered unless detected across multiple frames Salgado Manrique (2024).

Additionally, to account for intermittent signal loss, a track is not considered terminated unless no localizations are detected over two consecutive frames (approximately 0.6 s). The maximum distance for merging localizations into tracks was set to 200 nm, corresponding to the expected spatial uncertainty prior to merging step. Since duplicate localizations were already removed during the ThunderSTORM processing step, this threshold avoids overfitting.

After track formation, a distance threshold of 100 nm was used to merge tracks into molecular representations, reflecting the improved spatial precision achieved after merging localizations. Finally, for time series analysis, an interval of 50 s was selected to match the temporal analysis framework used in Dempsey et al. (2011), enabling direct comparison of results over time.

2 APP USAGE

The repository hosting the source code contains a Python based Streamlit application designed for the analysis and visualization of SOAC filament data and large scale STORM localization datasets. Instructions on how to install and run the application are provided within the repository.

Repository: `https://github.com/Alejandro1400/BlinkFusion`

This tutorial provides a concise overview of how to use the functionalities offered by the **BlinkFusion** application. The workflow is covered starting from the **Welcome** page—where relevant paths are

defined—through **Preprocessing**, **Batch Analysis** for both **Filament** and **STORM** pipelines, and finally the **Results Dashboard** functionalities.

Before proceeding, users should follow the installation instructions available in the repository. Throughout the application, **(?)** symbols provide contextual explanations of displayed elements and available functionalities.

2.1 Welcome

Upon launching the application, the user is presented with a welcome page (Fig. S1).

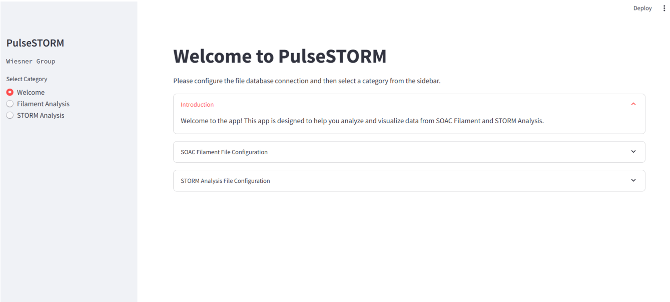

Figure S1. Welcome page of the BlinkFusion application.

2.1.1 SOAC Filament File Configuration

Users must first define the directory where images, analysis outputs, and results will be stored. For optimal performance, this path should be located on a **local hard drive**.

Next, a JSON parameter file must be configured to define both the **ridge detection** settings and the Region of Interest (ROI) selection criteria. An example configuration file is provided in `Data_access/ridge_detector_param.json`. Users are encouraged to follow the ridge detection tutorial and perform exploratory analysis in **ImageJ-Fiji** Schindelin et al. (2012) to determine appropriate parameter values Wagner et al. (2017). Further details on parameter definition are provided in Section 2.3.1.

Additionally, users must specify the path to the **SOAX batch analysis** parameter files and executable, example located in `Utils/SOAX`. Default values are recommended, as they provide a reliable baseline configuration Xu et al. (2015).

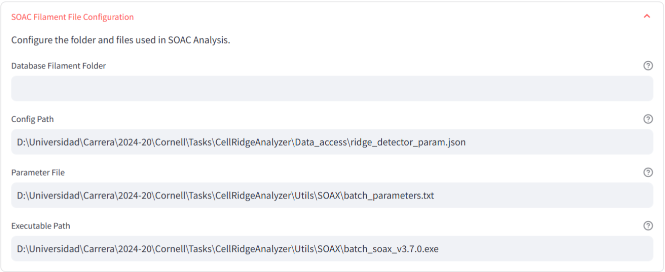

Figure S2. SOAC filament configuration interface.

2.1.2 STORM File Configuration

For STORM analysis, the **results folder** needs to be defined in this configuration section. The parameters for the STORM processing needs to be updated in `Data_access/storm_merge_param.json`. A more detailed explanation on this parameters is included in 2.3.2

2.1.3 Analysis Sidebar and Progress Indicators

On the left side of the interface, a sidebar provides access to either **Filament Analysis** or **STORM Analysis**.

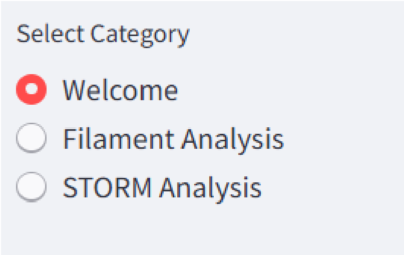

Figure S3. Application sidebar for selecting analysis modes.

When an analysis mode is selected, datasets from the corresponding directories—and, in the case of STORM, from the MongoDB database—are loaded into the Streamlit application.

The sidebar also displays progress indicators that reflect the current stage of the processing pipeline. For more detailed information, users can refer to the terminal output.

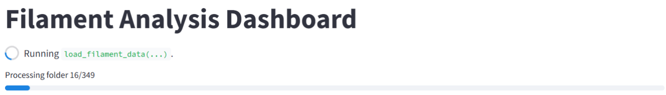

Figure S4. Progress indicators displayed during processing.

2.2 Preprocessing Data

The preprocessing step is designed to load images and datasets into the **Data Folder** and assign **metadata** for comparative analysis.

Users must define the directory containing the images to be processed. A hierarchical folder selection is supported, allowing the software to recursively traverse nested directories and load their contents. This approach should only be used when the parameters to be assigned, such as particle type, are consistent across all samples within the selected folders.

Certain metadata values are automatically extracted from **Zeiss microscope files** and **TIF metadata**, including image size. Additionally, the application allows users to define custom metadata fields, enabling structured organization of datasets for downstream analysis.

For **STORM processing**, users are encouraged to name image files using the convention:

`Image__angleX_laserX_expX_gainX`

where X represents the corresponding acquisition parameters for automatic metadata definition. Alternatively, they can be defined later using the **Database Metadata** or **Add New Metadata** options.

The preprocessing interface is divided into several tabs.

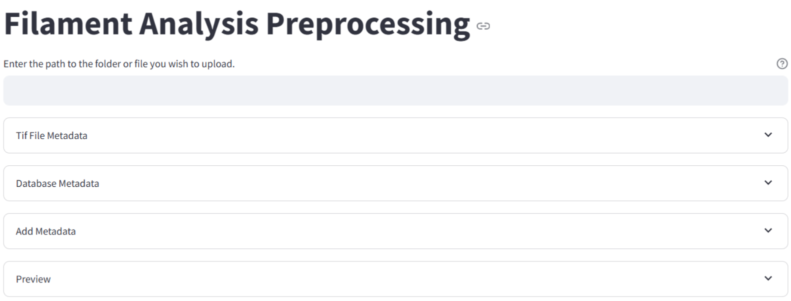

Figure S5. Preprocessing interface showing available tabs.

2.2.1 TIF or CZI File Metadata

In this tab, metadata relevant to the study, such as image size, laser settings, acquisition date, and number of frames, are automatically extracted from the input files. TIF files are used for filament analysis, while CZI or TIF files are used for STORM analysis.

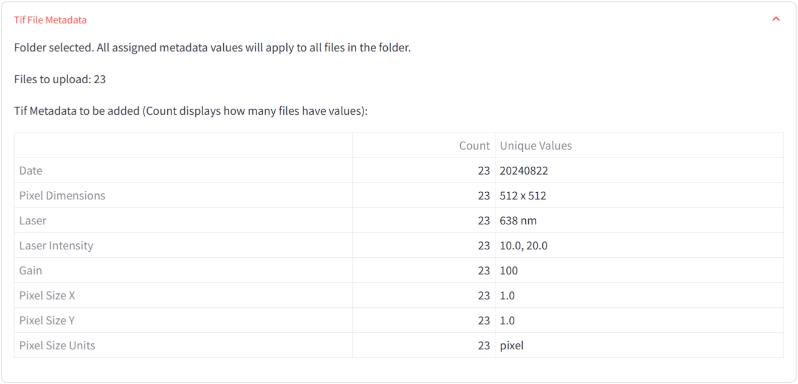
Tif File Metadata

Folder selected. All assigned metadata values will apply to all files in the folder.

Files to upload: 23

Tif Metadata to be added (Count displays how many files have values):

	Count	Unique Values
Date	23	20240822
Pixel Dimensions	23	512 x 512
Laser	23	638 nm
Laser Intensity	23	10.0, 20.0
Gain	23	100
Pixel Size X	23	1.0
Pixel Size Y	23	1.0
Pixel Size Units	23	pixel

Figure S6. Automatically extracted file metadata.

2.2.2 Database Metadata and Add New Metadata

Because metadata selection is user defined, the **Add Metadata** tab allows users to create new metadata entries for comparative analysis. Users specify the metadata name, variable type, and value.

Once a metadata value is created, it becomes available in the **Database Metadata** tab. To maintain consistency and improve efficiency, users can select from existing values, define new ones, or assign null values when appropriate. A folder level checkbox is also available. By default, files are sorted by date; however, users can define alternative organizational schemes of their choosing.

Finally, the **Preview** tab displays the resulting metadata assignments for the selected datasets. When files are uploaded, preprocessing is executed automatically. As a result, the selected directory is organized into a structured dataset containing one folder per dataset, a corresponding TIF image (CZI files are converted to

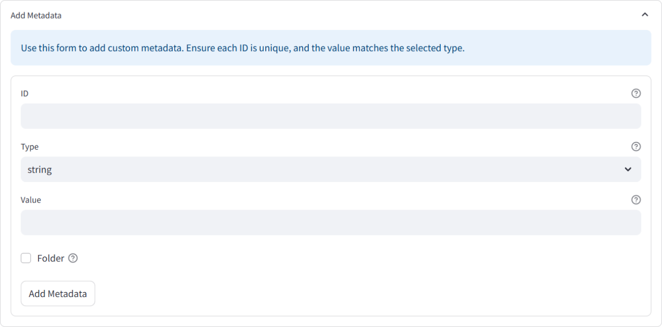

Figure S7. Interface for adding custom metadata.

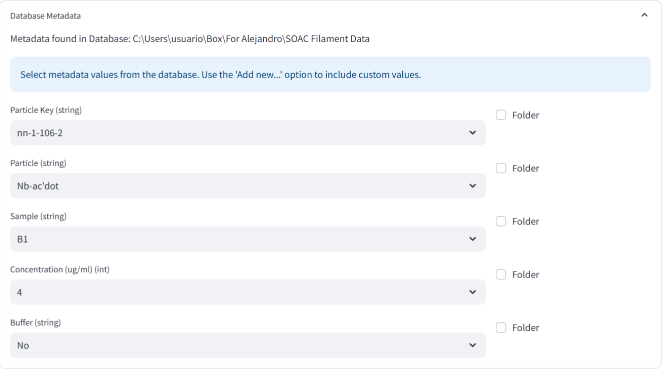

Figure S8. Database metadata selection interface.

TIF for STORM analysis), and embedded metadata. A progress bar indicates the preprocessing status for each uploaded file.

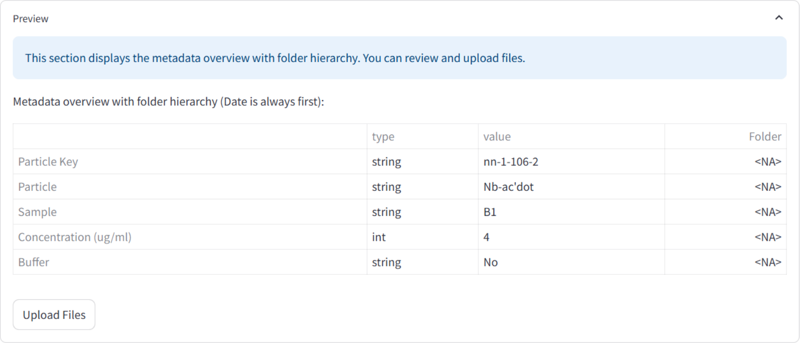

Figure S9. Preview of preprocessing results and progress tracking.

2.3 Batch Processing

During the batch processing stage, the software scans all dataset folders and identifies those eligible for analysis. Eligible datasets are defined as those that were added during preprocessing and have not yet undergone batch processing.

By default, all filament datasets are ready for batch processing immediately after preprocessing. In contrast, STORM datasets require prior processing using **ThunderSTORM** in ImageJ Ovesný et al. (2014);

Schindelin et al. (2012). Users must export localization results as CSV files with the suffix _locs.csv, which should be placed in the same directory as the corresponding image.

The batch processing interface provides detailed progress information for each dataset. This is particularly relevant for STORM localization analysis, which may require several minutes per dataset. An estimated remaining processing time is also displayed.

2.3.1 Filament Processing Configuration

For filament analysis, batch processing generates CSV files containing SOAC-derived statistics from high-quality regions identified through ridge detection Wagner et al. (2017), together with additional metrics obtained from SOAX analysis Xu et al. (2015). As described in Section 2.1.1, these parameters are defined through configuration files referenced in the Welcome Page.

Ridge detection parameters and ROI selection are estimated automatically, combined with a weighted ranking of candidate regions. This ensures that selected ROIs correspond to structurally meaningful and comparable filament regions across datasets. A detailed description of the parameter estimation procedure and ROI selection strategy is provided in Section 3.

The configuration file allows users to define ridge detection manual parameters (although it is encouraged to use the automatic processing), number of ROIs to divide the image in and the weights assigned to each ridge based metric. This enables flexible adaptation of the selection process to different datasets and experimental conditions. For manual tuning of these weights review section 3.2. The resulting ROIs are then processed using the SOAX framework, producing quantitative metrics such as filament length, intensity, contrast, and continuity, which are exported for downstream analysis.

2.3.2 STORM Processing Configuration

For STORM analysis, results are uploaded to a MongoDB database in a structured JSON format. To configure the database check the installation tutorial for the App in section 2.

The processing pipeline relies on a set of parameters that control the conversion of localizations into tracks, the merging of tracks into molecular representations, and the computation of time dependent metrics. These parameters are defined in the JSON configuration file, as defined in 2.1.2 and can be adjusted depending on the characteristics of the dataset.

The merge of localizations into tracks needs three parameters: the minimum number of frames required to define a valid track (`min_frames_locs_to_tracks`), the maximum number of allowed frame gaps between consecutive detections (`max_gaps_locs_to_tracks`), and the maximum spatial distance for associating localizations (`max_distance_merge_localizations`). Together, these parameters determine how robustly individual blinking events are grouped into continuous trajectories, balancing sensitivity to short-lived events and tolerance to intermittent signal loss.

Once tracks are generated, they are merged into molecular representations using a spatial threshold defined by `max_distance_merge_molecules` which we suggest to be lower or same to the distance to merge localizations since the exact position should be more precise when comparing tracks. This parameter controls the extent to which nearby tracks are considered to originate from the same molecule, and therefore directly influences the spatial aggregation of localization events.

Finally, time-dependent analysis is performed by grouping molecular events into temporal bins defined by the parameter `time_series_interval_seconds`. This interval determines the temporal resolution of the resulting time series and should be selected according to the desired analysis.

Users are encouraged to explore their effect on the results and select values that best reflect the expected temporal continuity and spatial organization of the underlying molecular structures.

2.4 Dashboard Comparative Analysis

The analysis workflow is similar for both filament and STORM datasets. The main difference is that the STORM dashboard includes more detailed single image analysis. Due to the stochastic nature of STORM data, individual datasets should be carefully examined, while comparative analysis serves as an initial exploratory tool.

2.4.1 Filtering Step

In the **Filter Metadata Options** section, users select specific metadata values to filter datasets, such as acquisition date, particle type, or experimental conditions.

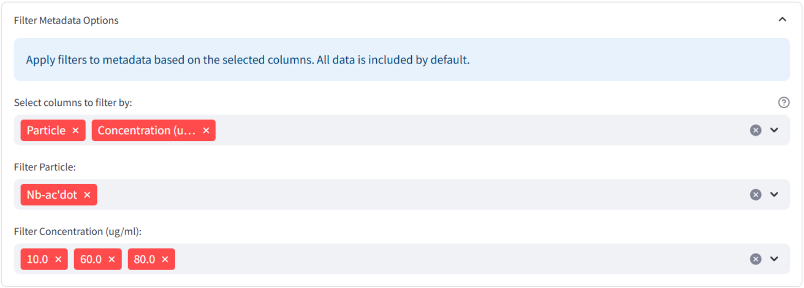

Figure S10. Metadata based dataset filtering interface.

The **Filter Data** section is specific to filament analysis and allows users to exclude filaments that do not meet predefined criteria. For example, if longer filaments are considered more reliable, users can apply a minimum length threshold. The interface also displays the number of remaining filaments after filtering, ensuring that a sufficient number of data points is retained for comparative analysis. This functionality was extensively used during research and validation to remove outliers.

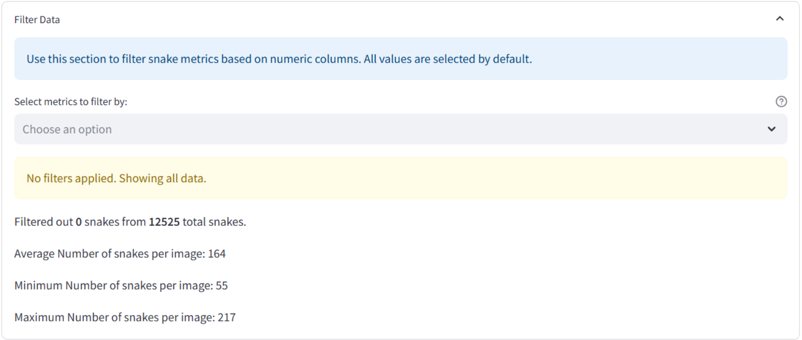

Figure S11. Filament level data filtering interface.

2.4.2 Analysis

The data analysis section displays mean values and standard deviations for quantitative metrics. Results are grouped hierarchically based on user selected metadata.

These results can be exported as tables for rapid interpretation and inclusion in presentations or reports.

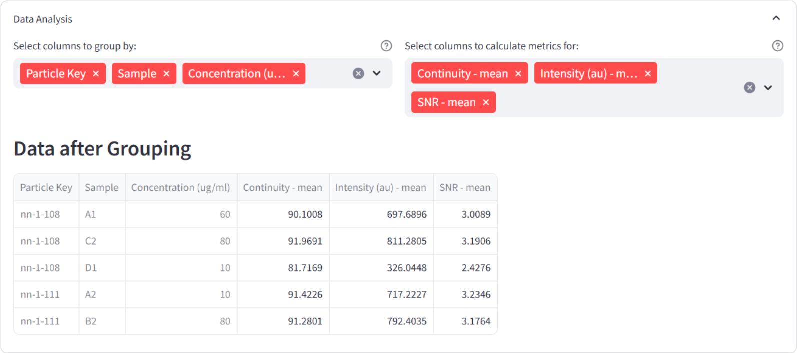

Particle Key	Sample	Concentration (ug/ml)	Continuity - mean	Intensity (au) - mean	SNR - mean
nn-1-108	A1	60	90.1008	697.6896	3.0089
nn-1-108	C2	80	91.9691	811.2805	3.1906
nn-1-108	D1	10	81.7169	326.0448	2.4276
nn-1-111	A2	10	91.4226	717.2227	3.2346
nn-1-111	B2	80	91.2801	792.4035	3.1764

Figure S12. Quantitative data analysis and summary statistics.

The Comparative Graphs Analysis section enables visualization of the same quantitative results using multiple plot types, including histograms, line plots, bar charts, and violin plots. Users can generate comparative figures efficiently and export them for further use.

2.5 Single Image Dashboard Analysis for STORM

STORM analysis includes a dedicated **single-image dashboard**. As in previous steps, users first filter and select the dataset to be analyzed. Once selected, detailed quantitative metrics are displayed.

2.5.1 Time-Series Analysis

This section presents relevant blinking metrics, such as duty cycle, survival fraction, and intensity over time, computed in time windows of 50 seconds (based on the selected parameters).

Both graphical plots and corresponding tables are provided, enabling rapid inspection and export of time series results.

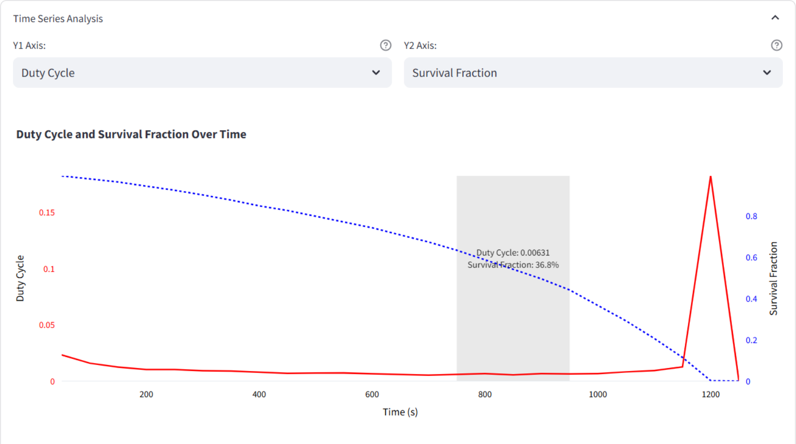

Figure S13. Time series analysis of blinking metrics.

2.5.2 Histogram Analysis

Histogram analysis presents key blinking metrics using multiple analysis modes:

- **By molecule**, where values are grouped by the mean per molecule
- **By switching cycle**, where each on/off cycle is weighted equally

Users can customize the number of bins, restrict analysis to quasi-equilibrium populations (where the duty cycle is most stable), and remove outliers. These options are particularly important for mitigating artifacts and obtaining representative population level estimates given the stochastic nature of STORM data. Exporting histogram values is also supported.

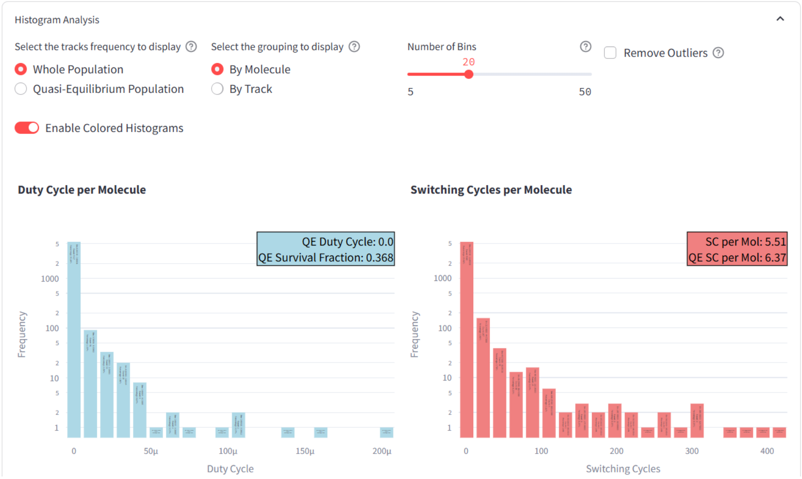

Figure S14. Histogram-based analysis of blinking metrics.

2.5.3 Blinking Classification

The blinking classification tool displays the intensity profile of individual molecules over time and assigns a classification based on the number of on/off switching events and the duty cycle Binkley and Griffin (2021).

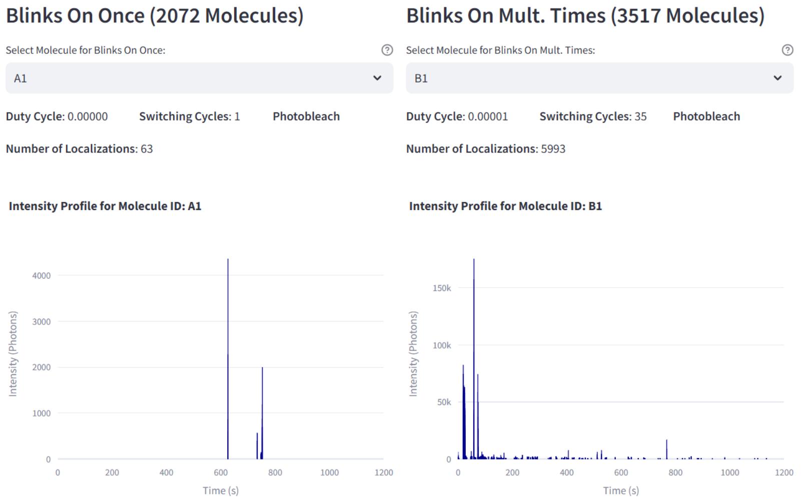

Figure S15. Blinking classification of individual molecules.

3 RIDGE DETECTION PARAMETER ESTIMATION

The ridge detection software Wagner et al. (2017), available in Fiji (ImageJ), is a practical tool for filament analysis. In this work, it is used to identify image regions suitable for homogeneous comparison of tubulin labeling.

Although the plugin supports automated execution, its performance strongly depends on the correct selection of parameters. Understanding how these parameters are defined is therefore essential, both for manual analysis and for the development of automated estimation strategies.

This section is divided into two parts. First, we describe the manual workflow used to estimate ridge detection parameters, including the definition of line width and contrast values. Then, we present the automated parameter estimation and ROI selection approach, where parameters are derived from image statistics and regions are ranked using weighted metrics.

The results obtained from the manual procedure were compared with the automated approach, showing that parameter estimation on smaller regions of interest (ROIs) yields comparable results while significantly reducing manual workload. When combined with weighted ROI selection, the parameter estimation does not need to be exact, as the final quantitative accuracy is achieved through the optimization process used for filament analysis with the SOAX software Xu et al. (2015). Additional details on the automated batch processing are provided in Section 2.3.1.

3.1 Manual Workflow

3.1.1 Preprocessing Workflow

1. Load the image into ImageJ. If the input is a video stack, compute the average projection via:
   `Image → Stacks → Z Project → Average Intensity.`
2. **Optional:** If prompted about incompatible formats, convert the image to 8-bit:
   `Image → Type → 8-bit.`
3. Before running the Ridge Detection plugin, estimate the optional parameters, which determine the mandatory parameters.

3.1.2 Parameter Definitions

The ridge detection parameters are computed as sigma, upper threshold and lower threshold from the filament width and contrast values as follows:

$$\sigma = \frac{w}{2\sqrt{3}} + 0.5 \tag{S1}$$

$$T_U = \left\lfloor 0.17 \cdot \frac{2 \cdot b_{\text{upper}}}{\sqrt{2\pi}\,\sigma^3} \cdot \exp\left(-\frac{\left(\frac{w}{2}\right)^2}{2\sigma^2}\right)\right\rfloor \tag{S2}$$

$$T_L = \left\lfloor 0.17 \cdot \frac{2 \cdot b_{\text{low}}}{\sqrt{2\pi}\,\sigma^3} \cdot \exp\left(-\frac{\left(\frac{w}{2}\right)^2}{2\sigma^2}\right)\right\rfloor \tag{S3}$$

3.1.3 Practical Estimation Procedure

1. Select a region with high signal-to-noise ratio (SNR), typically near the image edges.

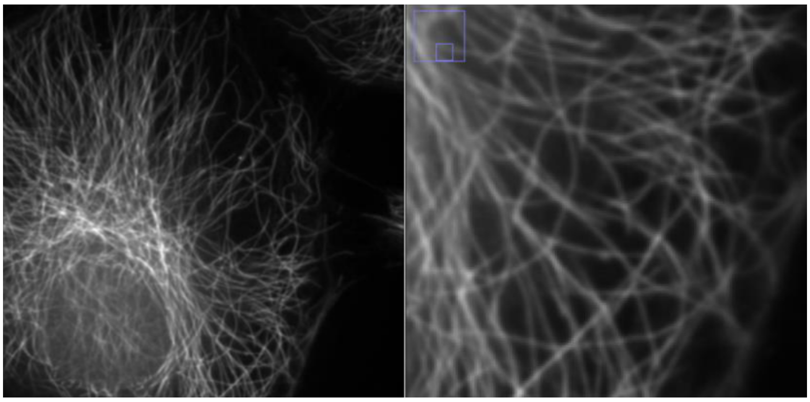

Figure S16. Zoomed high-SNR region.

2. **Line width:** Zoom to pixel resolution and draw a line across the filament width using the `Straight` tool.

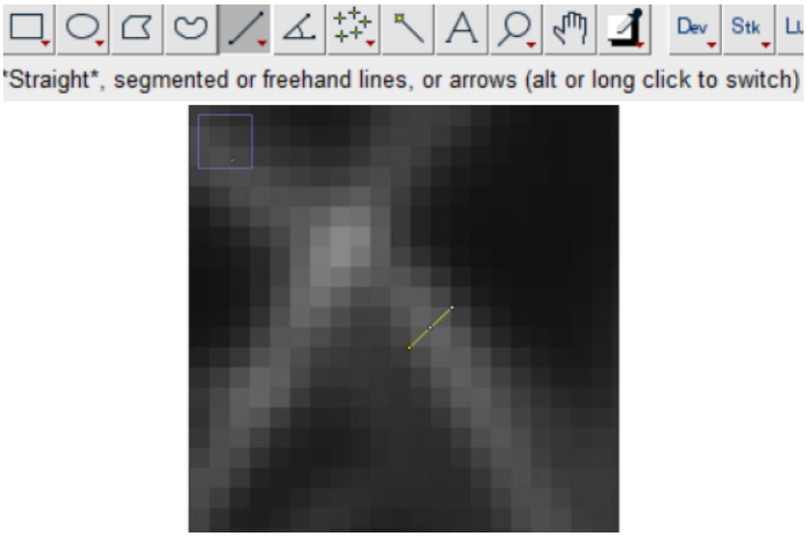

Figure S17. Filament width measurement.

Measure using `Ctrl+M` or `Analyze` $\rightarrow$ `Measure`. The reported length corresponds to w.

	Area	Mean	Min	Max	Angle	Length
1	4	79.730	64.417	91.956	43.831	2.888

Figure S18. Measured width values.

3. **High contrast (b_{upper}):** Draw a line through the filament center and use the mean intensity.
4. **Low contrast (b_{low}):** Draw a line near the filament edge (still within the structure, not background) and measure the mean intensity.

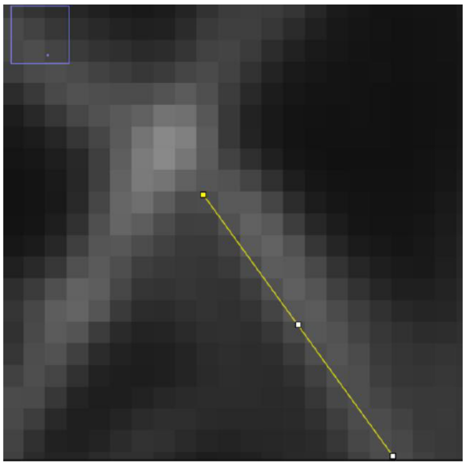

	Area	Mean	Min	Max	Angle	Length
1	16	78.605	74.722	82.015	-53.996	14.885

Figure S19. High contrast measurement.

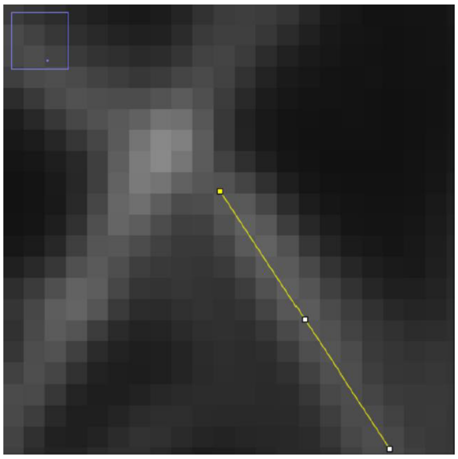

	Area	Mean	Min	Max	Angle	Length
1	16	84.831	73.882	95.294	-56.811	14.538

Figure S20. Low contrast measurement.

3.1.4 Ridge Detection Execution

1. Return to the selected region and open:
   `Plugins → Ridge Detection`.
2. Input the estimated parameters. The mandatory parameters will be computed automatically.
3. Ensure the same configuration settings, with the values obtained through the process, as shown below.
4. Click `Preview` to visualize detected filaments and junction points.

3.1.5 Parameter Tuning

Initial results may miss low-intensity filaments. Adjust parameters iteratively, prioritizing:

- Increasing filament detection coverage
- Avoiding excessive junction points (indicative of overfitting)

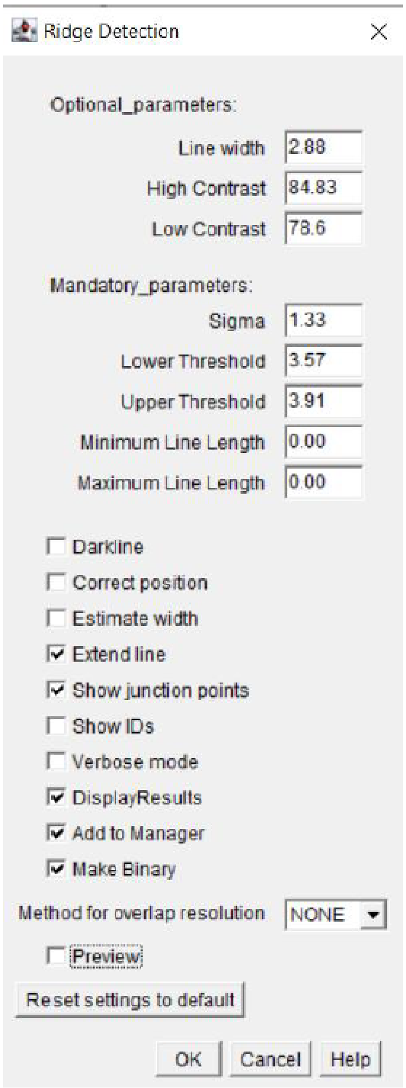

Figure S21. Ridge detection parameters.

Excessive junction clustering suggests overfitting:

Empirically, parameters typically require only minor adjustments, often slight reductions.

3.1.6 Final Validation

After tuning in the zoomed region, evaluate performance on the full image and perform final adjustments if necessary.

3.2 Ridge Detection and ROI Automated Process

To automatically estimate ridge detection parameters, we implemented a method based on image statistics. For each ROI, the mean intensity and standard deviation are computed Wagner et al. (2017). The mean intensity defines a lower contrast baseline, while the standard deviation determines a higher contrast level. These values, together with a user-defined line width, are used to compute the plugin parameters `Sigma`, `Lower_Threshold`, and `Upper_Threshold`. This ensures that the filter adapts to the expected filament width and local signal variations when running the ridge detection algorithm Steger (1998).

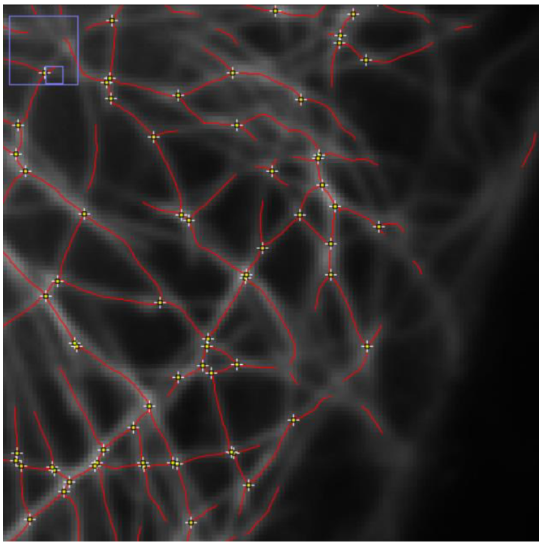

Figure S22. Preview of detected filaments.

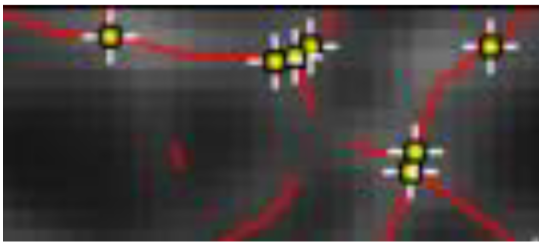

Figure S23. Overfitting with excessive junctions.

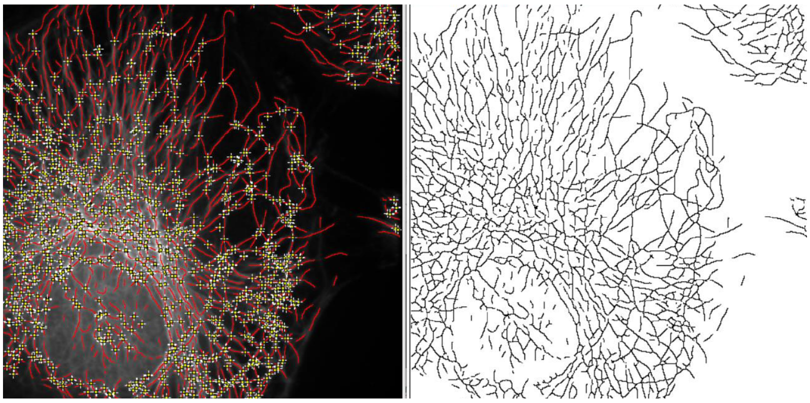

Figure S24. Final filament detection result.

Alternatively, these parameters can be manually defined, as described in 2.3.1. However, improved parameter estimation is achieved when this process is performed on individual ROIs, since different regions of the image may exhibit significant variations in intensity and contrast. In practice, manually analyzing multiple ROIs (e.g., 16 regions per image across several datasets) results in a substantial workload and increases the likelihood of human error.

To address this limitation, ROI selection is automated using a weighted combination of metrics computed from the ridge detection results. The weights assigned to each metric, together with the number of selected

regions, are defined in the configuration file. Among the available metrics, particular importance is given to *Number of Ridges*, *Ridge/Junction Ratio*, and *Mean Length*. These criteria prioritize regions with a high filament density, minimal overlap (reducing artificial intensity increases and 3D projection effects), and longer filament structures, enabling more reliable comparisons Li et al. (2009).

We recommend using the automated processing described in 2.3.1, especially when comparing multiple images. In such cases, users are encouraged to evaluate ROI selection under different weight configurations and manually inspect the resulting ROIs. This can be done by reviewing the generated TIF files for each selected region and comparing them with the original images to ensure that the selected regions correspond to meaningful structures. For example, in tubulin datasets, optimal ROIs typically correspond to cell edges, where overlap and background noise are reduced, enabling more homogeneous filament comparisons Schvartz et al. (2017); Martinez-Sanchez et al. (2013).

Overall, this automated framework enables consistent parameter estimation and ROI selection across large datasets, significantly reducing manual intervention while preserving sensitivity to local image variations. By combining statistical parameter estimation with weighted ROI ranking, the method selects regions that are both structurally meaningful and quantitatively comparable. These ROIs serve as input for downstream filament segmentation and quantitative analysis, supporting scalable and reproducible assessment of labeling efficiency across experimental conditions.

4 THUNDERSTORM PROCESSING USAGE

The use of ThunderSTORM Ovesný et al. (2014) is required as a manual step after submitting STORM datasets and before batch processing. This tutorial describes the manual stage of the blinking statistics workflow: starting from a stack of STORM microscopy images, we extract a list of localizations using ThunderSTORM in ImageJ.

4.1 Prerequisites and Installation

1. **Install ImageJ (or Fiji)**
   Download the latest version from:
   `https://imagej.net` or `https://fiji.sc`.
2. **Download ThunderSTORM plugin**
   Obtain the latest `.jar` file from the official repository:
   `https://github.com/zitmen/thunderstorm`

   Additional documentation and tutorials are available at:
   `https://github.com/zitmen/thunderstorm/wiki/Tutorials`
3. **Install the plugin**
   - Copy the downloaded `.jar` file into the `plugins/` directory of ImageJ or Fiji.
   - Restart ImageJ to activate the plugin.

4.2 Processing Workflow

Once installed, each preprocessed TIFF file can be analyzed as follows:

Processing workflow. Each preprocessed TIFF file is analyzed in ThunderSTORM as follows.

First, open the image via `File` → `Open` and select *Open as HyperStack*. Visibility can be improved using `Process` → `Enhance Contrast`.

Then, run the analysis through `Plugins` → `ThunderSTORM` → `Run Analysis`. Default parameters generally provide a good starting point (Fig. S25), but can be adjusted depending on the dataset, for example by tuning the sub-pixel localization according to particle size or enabling multi-emitter fitting for dense samples.

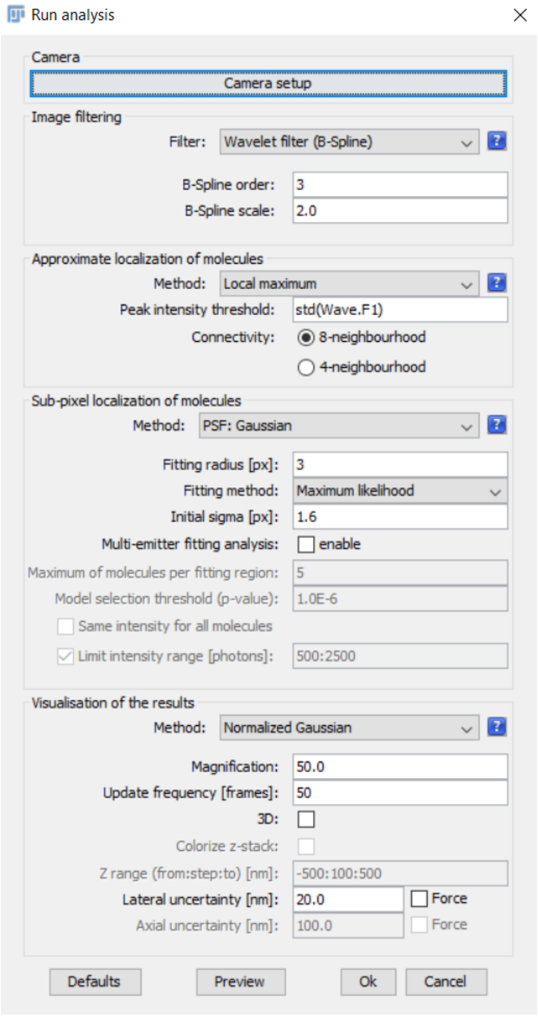

Figure S25. ThunderSTORM parameter settings.

The output consists of localization data including coordinates, intensity, and uncertainty (Fig. S26). Post-processing should include removal of duplicate detections to avoid false positives and drift correction, particularly for long acquisitions where sample motion may introduce artifacts. Finally, results are exported via `Export` → `CSV file` and saved in the same directory as the original TIFF image using the naming convention `filename_locs.csv`.

Maintaining consistent naming conventions between TIFF and CSV files facilitates downstream automation and integration with BlinkFusion.

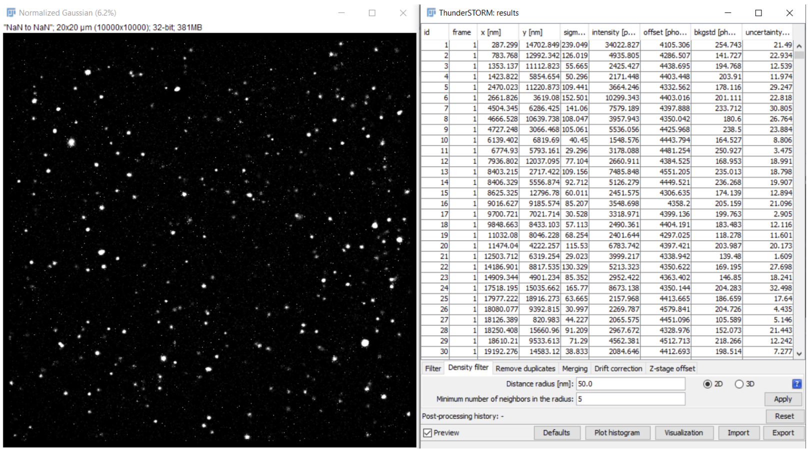

Figure S26. Localization results output.

REFERENCES

Binkley, K. E. and Griffin, C. (2021). Imaging analysis of photoswitching fluorophores using single-molecule microscopy. *SMU J. Undergrad. Res.* 6, 1. doi:10.25172/jour.6.2.1

Dempsey, G. T., Vaughan, J. C., Chen, K. H., Bates, M., and Zhuang, X. (2011). Evaluation of fluorophores for optimal performance in localization-based super-resolution imaging. *Nat. Methods* 8, 1027–1036. doi:10.1038/nmeth.1768

Křížek, P., Raška, I., and Hagen, G. M. (2011). Minimizing detection errors in single molecule localization microscopy. *Opt. Express* 19, 3226–3235. doi:10.1364/OE.19.003226

Li, H., Shen, T., Vavylonis, D., and Huang, X. (2009). Actin filament tracking based on particle filters and stretching open active contour models. In *Med. Image Comput. Comput. Assist. Interv.* vol. 12, Pt. 2, 673–681. doi:10.1007/978-3-642-04271-3-82

Martinez-Sanchez, A., Garcia, I., and Fernandez, J.-J. (2013). A ridge-based framework for segmentation of 3d electron microscopy datasets. *J. Struct. Biol.* 181, 61–70. doi:10.1016/j.jsb.2012.10.002

Ovesný, M., Křížek, P., Borkovec, J., Švindrych, Z., and Hagen, G. M. (2014). Thunderstorm: A comprehensive imagej plugin for palm and storm data analysis and super-resolution imaging. *Bioinformatics* 30, 2389–2390. doi:10.1093/bioinformatics/btu202

Salgado Manrique, A. (2024). *Enhancing aC'dots analysis through STORM imaging: development of advanced computational tools for super-resolution microscopy*. Undergraduate thesis, Universidad de Los Andes

Schindelin, J., Arganda-Carreras, I., Frise, E., Kaynig, V., Longair, M., Pietzsch, T., et al. (2012). Fiji: An open-source platform for biological-image analysis. *Nat. Methods* 9, 676–682. doi:10.1038/nmeth.2019

Schvartz, T., Aloush, N., Goliand, I., Segal, I., Nachmias, D., Arbely, E., et al. (2017). Direct fluorescent-dye labeling of -tubulin in mammalian cells for live cell and superresolution imaging. *Mol. Biol. Cell* 28, 2747–2756. doi:10.1091/mbc.E17-03-0161

Shimizu, Y. (2007). The wavelet transform in signal and image processing. In *Frontiers in Computing Technologies for Manufacturing Applications* (London, U.K.: Springer), Springer Series in Advanced Manufacturing. doi:10.1007/978-1-84628-955-2_5

Steger, C. (1998). An unbiased detector of curvilinear structures. *IEEE Trans. Pattern Anal. Mach. Intell.* 20, 113–125. doi:10.1109/34.659930

Wagner, T., Hiner, M., and Xraynaud (2017). thorstenwagner/ij-ridgedetection: Ridge detection 1.4.0 doi:10.5281/zenodo.845874. Zenodo, version 1.4.0

Xu, T., Vavylonis, D., Tsai, F. C., Koenderink, G. H., Nie, W., Yusuf, E., et al. (2015). Soax: A software for quantification of 3d biopolymer networks. *Sci. Rep.* 5, 9081. doi:10.1038/srep09081
